# Revisiting Vibrational Spectroscopy to Tackle the Chemistry of Zr_6_O_8_ Metal-Organic Framework Nodes

**DOI:** 10.1021/acsami.2c04712

**Published:** 2022-05-31

**Authors:** Ignacio Romero-Muñiz, Carlos Romero-Muñiz, Isabel del Castillo-Velilla, Carlo Marini, Sofía Calero, Félix Zamora, Ana E. Platero-Prats

**Affiliations:** † Departamento de Química Inorgánica, Facultad de Ciencias, Universidad Autónoma de Madrid, Campus de Cantoblanco, 28049 Madrid, Spain; ‡ Departamento de Física Aplicada I, Universidad de Sevilla, E-41012 Seville, Spain; § CLAESS beamline, ALBA Synchrotron, Cerdanyola del Vallès 08290, Spain; ∥ Materials Simulation & Modelling, Department of Applied Physics, Eindhoven University of Technology, 5600MB Eindhoven, The Netherlands; ⊥ Condensed Matter Physics Center (IFIMAC), Universidad Autónoma de Madrid, Campus de Cantoblanco, 28049 Madrid, Spain; # Instituto de Investigación Avanzada en Ciencias Químicas de la UAM, Universidad Autónoma de Madrid, Campus de Cantoblanco, 28049 Madrid, Spain

**Keywords:** zirconium metal-organic frameworks, density functional theory calculations, vibrational spectroscopy, local structure, 1,3-dipolar cycloaddition

## Abstract

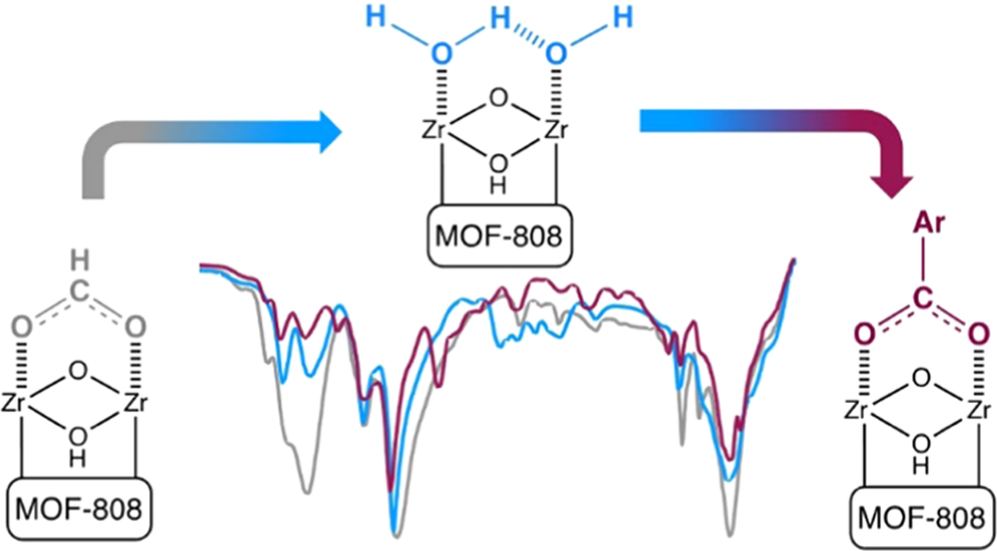

The metal-organic framework MOF-808 contains Zr_6_O_8_ nodes with a high density of vacancy sites, which can incorporate carboxylate-containing functional groups to tune chemical reactivity. Although the postsynthetic methods to modify the chemistry of the Zr_6_O_8_ nodes in MOFs are well known, tackling these alterations from a structural perspective is still a challenge. We have combined infrared spectroscopy experiments and first-principles calculations to identify the presence of node vacancies accessible for chemical modifications within the MOF-808. We demonstrate the potential of our approach to assess the decoration of MOF-808 nodes with different catechol–benzoate ligands. Furthermore, we have applied advanced synchrotron characterization tools, such as pair distribution function analyses and X-ray absorption spectroscopy, to resolve the atomic structure of single metal sites incorporated into the catechol groups postsynthetically. Finally, we demonstrate the catalytic activity of these MOF-808 materials decorated with single copper sites for 1,3-dipolar cycloadditions.

## Introduction

The development of metal-organic frameworks (MOFs) offers the opportunity to arrange building blocks of molecular nature to afford crystalline open scaffolds with permanent porosity.^1,2^ MOFs have hollowed crystal structures with different symmetries, which can be tailored by choosing molecular components with target geometries and connectivity. Their tunable textural and structural properties have pointed MOFs as very promising systems for gas storage and separation,^3,4^ pollutant removal,^5^ and catalytic^6−8^ and biological applications^9^—all processes where the diffusion of molecules through the MOF pores and surfaces is key for the final performance of the material. Among the large variety of chemistry explored to construct these porous architectures, the family of Zr(IV)-MOFs are of particular interest due to their thermal and chemical stability, which expands the potential applications in a wide range of conditions.^10^ In this context, [Zr_6_O_8_H_4_]^12+^ nodes connected through carboxylate organic linkers can afford to obtain 12-, 8-, or 6-connected frameworks, UiO-66,^11^ NU-1000,^12^ and MOF-808^13^ being the archetypal ones, respectively.

**Figure 1:**
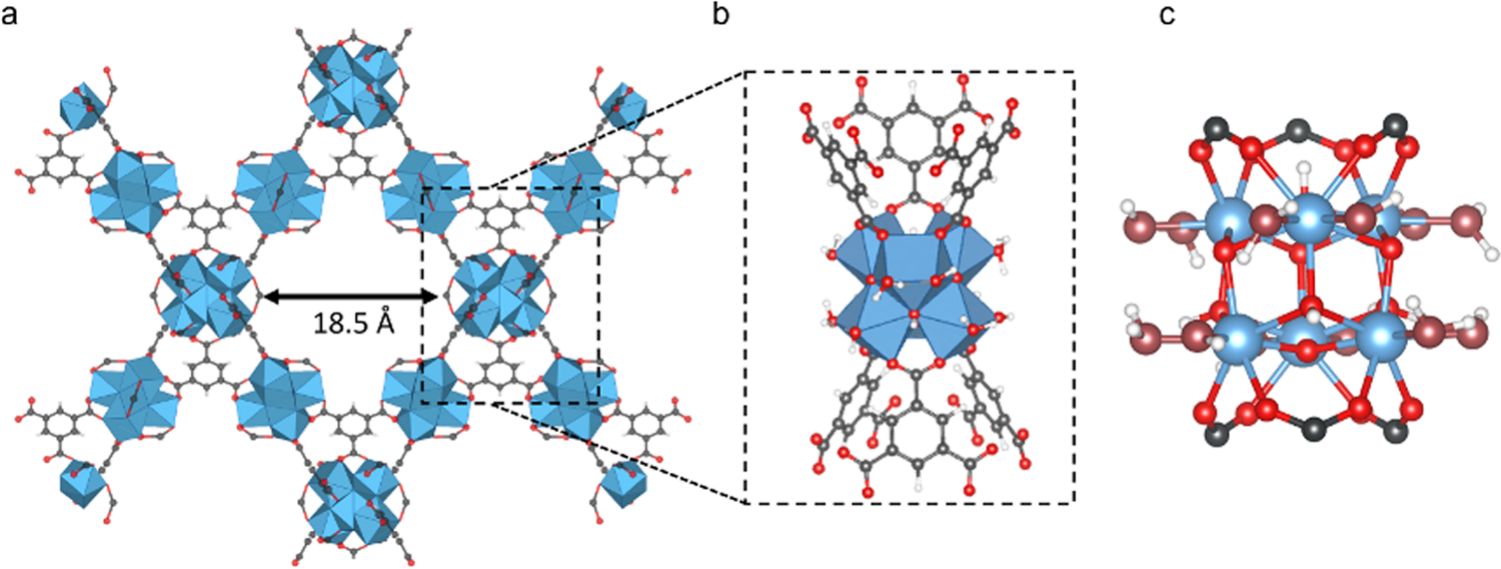
Pore window of MOF-808 (a) and details of the unsaturated [Zr_6_O_8_H_4_]^12+^ node (b). Representation of the zirconia nodes highlighting the labile −OH and −H_2_O groups in dark red (c). Carbon: black, oxygen: red, zirconium: blue, and hydrogen: white.

In particular, the structure of MOF-808 is built from linking highly unsaturated Zr–oxo nodes with benzene-1,3,5-tricarboxylate (BTC) ligands, resulting in a cubic lattice with hexagonal mesopores of ca. 20 Å in diameter (Figure 1a).^13^ The porous nature of MOF-808 has been exploited in both gas- and liquid-phase applications.^14−16^ Interestingly, MOF-808 has a remarkable potential for chemical modification arising from the low saturation of the inorganic clusters. The as-synthesized MOF-808 material contains a total of six formate ligands located within the equatorial positions of the zirconia node, which can be transformed into defect sites by exchange with labile ligands (e.g., hydroxo/aquo) through chemical activation (Figure 1c). Thus, a total of 12 potential open metal sites located within the equatorial plane of the Zr–oxo nodes in MOF-808 can be envisaged for the grafting of a variety of functional groups, such as amino acids,^17^ phosphates,^18^ sulfates,^19^ sulfamates,^20^ and metal complexes.^21^ This postsynthesis strategy opens a new chemical scenario for the incorporation of new functionalities at specific locations within the MOF-808 nodes.^22−24^

One of the main characterization challenges regarding the chemical modification of the zirconia nodes in MOFs is to assess the exchange of labile groups by functional ligands. Indeed, the zirconia nodes and the added ligands can interact either by supramolecular host–guest interactions or by coordination bonds at the available open metal sites. In this regard, infrared spectroscopy is a suitable technique to characterize both chemical bonds and interactions in materials.^25^ In particular, Fourier-transform infrared spectroscopy (FTIR) has been used to assess the chemical modifications of the MOF-808 nodes with a variety of functional groups, including both organic and inorganic.^26,27^ However, the most common use of this technique is the monitoring of signals associated with target functional groups characteristic of the new molecules added that are not present in the MOF framework itself.^28–30^ Recently, FTIR combined with computational studies has been applied to assess the tuning of defect sites in Zr-MOFs with methoxy/ethoxy groups.^31^ However, there are numerous examples of MOF modification (i.e., aromatic carboxylate), where the added ligands are similar to the MOF linkers, thereby making FTIR data ambiguous and difficult to interpret.

In particular, catechols are interesting aromatic ligands with coordinating groups, for which their noninnocent redox activity has been widely explored in metal complexes.^32^ These types of ligands (and their analogues containing sulfur and nitrogen) have been used as linkers for constructing MOFs with conductive properties.^33,34^ On the other hand, catechol groups have been demonstrated to stabilize copper sites in a low oxidation state within a zirconia UiO-type material.^35^ In this context, the use of benzoate ligands containing catechol groups could be an excellent approach to expand the catalytic applications of MOF-808 through the postsynthetic modification of the Zr–oxo cluster. However, the similar structure of benzoate moieties compared to that of the BTC linker makes their complete characterization by IR spectroscopy challenging. In this context, the use of first-principles calculations to simulate all of the vibrational modes of a given MOF could represent a powerful characterization approach not only to flag chemical modification but also to retrieve full structural information of the material at the local scale.

We report a combination of experimental FTIR spectroscopy and density functional theory (DFT) calculations for the incisive characterization of MOF-808 modified with different isomers of a catechol ligand such as the dihydroxybenzoic acid (DHBA). The catechol moieties within DHBA ligands have been used as scaffolds for metalation reactions using a variety of transition metals, as demonstrated by X-ray absorption spectroscopy (XAS) and pair distribution function (PDF) analyses. In the case of copper, the reactivity of the isolated sites bonded to the catechol groups has been explored for the catalytic regioselective formation of 1,4-disubstituted 1,2,3-triazole derivatives, showing different performances depending on the relative position of the catechol groups within the MOF-808 nodes.

## Results and Discussion

MOF-808 was synthesized and thermally activated using reported procedures (Supporting Information, Section S1).^19^ Le Bail refinements of powder X-ray diffraction (PXRD) data collected on the MOF samples demonstrated the unique presence of the Bragg peaks corresponding to the MOF-808 phase (Supporting Information, Section S2). Proton nuclear magnetic resonance (^1^H NMR) of the digested samples (Supporting Information, Section S9) and TGA indicated a chemical formula for the material of [Zr_6_O_8_H_4_](BTC)_2_(formate)_6_ (MOF-808-F). This result agrees with the presence of the Zr–oxo node fully saturated with formate ligands used in the synthesis as modulators. Nitrogen isotherms collected on MOF-808 activated at 130 °C for 16 h indicated a Brunauer–Emmett–Teller (BET) surface area of 1375 cm^2^ g^–1^ and a pore width of 18 Å (Supporting Information, Section S4), in accordance with the porous structure of the material. The analogous MOF-808 material without formates (MOF-808-P) was synthesized as an activated form of the framework. The formate ligands within the equatorial plane of the Zr_6_O_8_ nodes can be fully removed by washing with methanol,^36^ as corroborated by ^1^H NMR on the digested samples (Supporting Information, Section S9). MOF-808-P showed an enhanced BET surface area of 1712 cm^2^ g^–1^ and a pore width of 20 Å, demonstrating the retaining of the porous structure after the chemical activation of the zirconia nodes.

**Figure 2:**
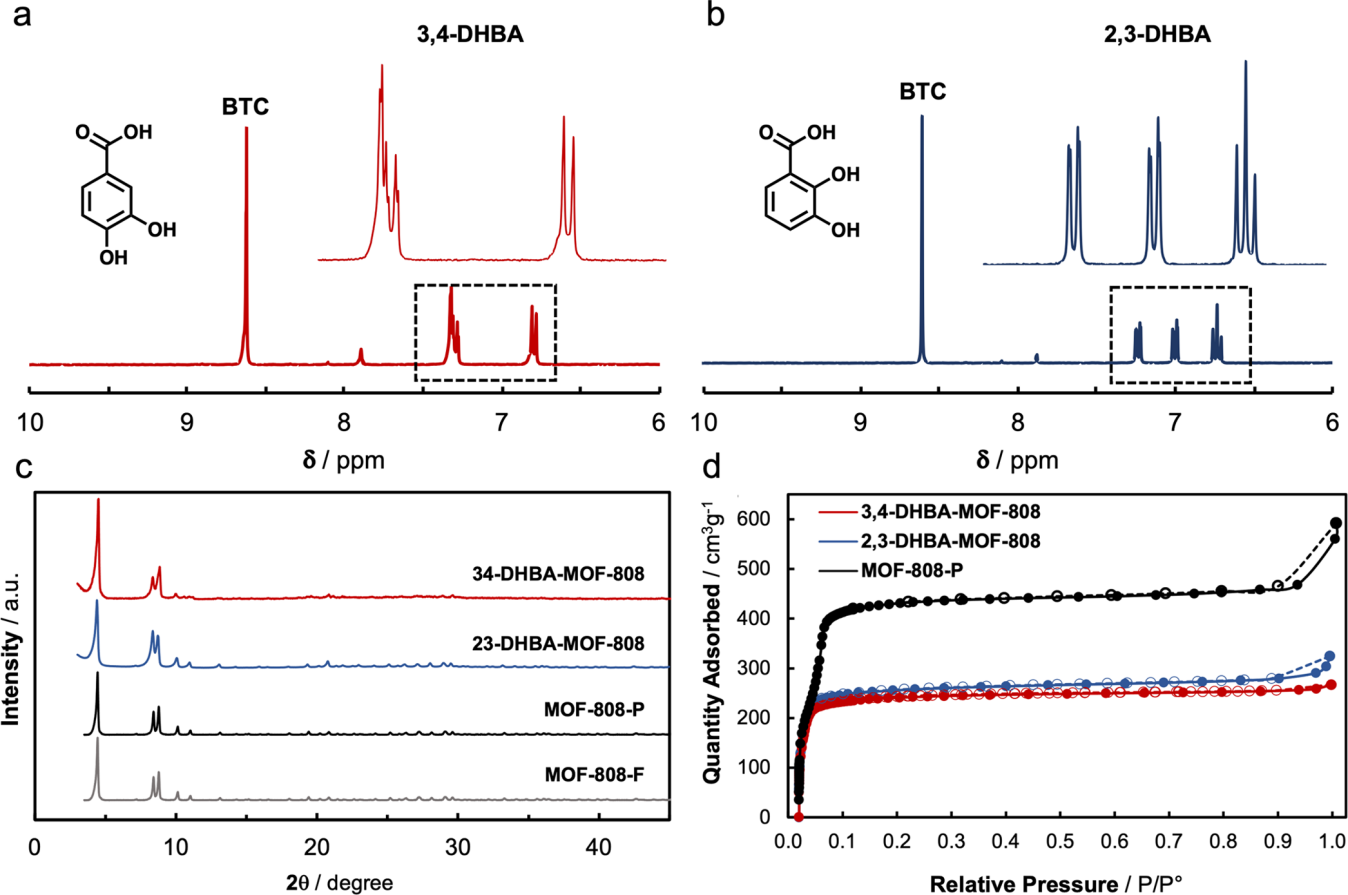
^1^H NMR spectra of the DHBA–MOF-808 systems (a and b). PXRD data (c) and N_2_ isotherms at 77 K of the DHBA–MOF-808 materials (d).

**Figure 3:**
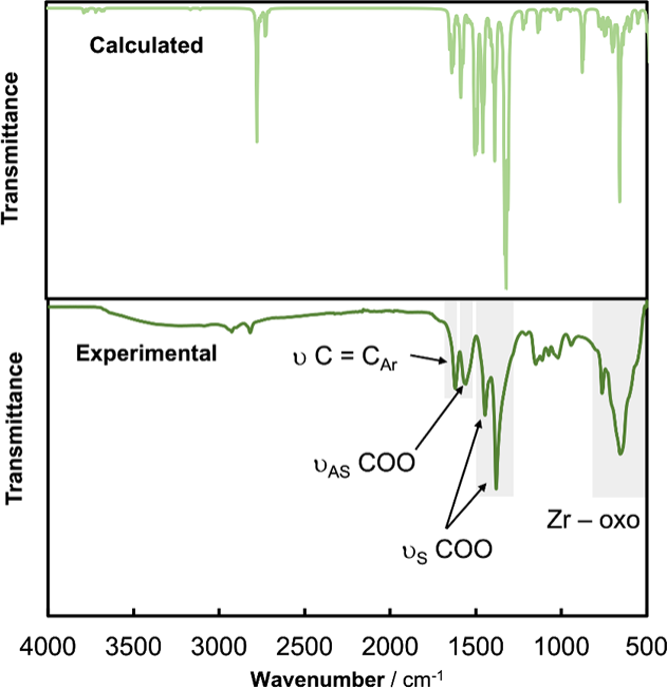
Comparison of the experimental and DFT-calculated IR spectra for MOF-808-P.

**Scheme 1:**
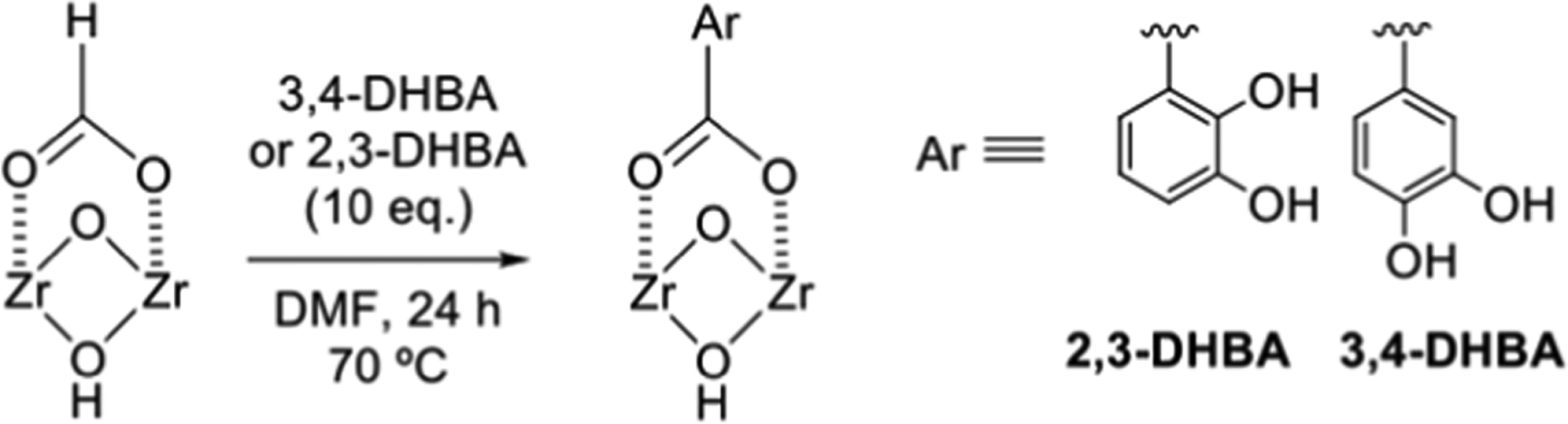
Ligand Substitution Reaction on a [Zr_6_O_8_H_4_]^12+^ Node of MOF-808 between the Dihydroxybenzoate and Formate Ligands

The MOF-808-F was modified with two catechol–benzoate ligands (i.e., 2,3- and 3,4-dihydroxybenzoic acid named 2,3- and 3,4-DHBA, respectively) (Scheme 1) to explore the capability for chemical modification of the unsaturated node. The incorporation of the DHBA ligands into MOF-808 was performed by solvent-assisted ligand incorporation methods.^22^ Thus, MOF-808-F samples were treated with a solution containing 1.7 equiv of DHBA per Zr in DMF at 70 °C for 24 h. The resulting material was activated with methanol to remove the remaining formate ligands within the MOF. Using this methodology, two new materials were obtained: 2,3- and 3,4-DHBA–MOF-808. PXRD data collected on the activated systems indicated that the average framework is retained after chemical modification (Figure 2c). ^1^H NMR analyses on the digested DHBA–MOF-808 materials indicated the incorporation of 3-DHBA ligands per MOF node, resulting in chemical composition [Zr_6_O_8_H_4_](BTC)_2_(DHBA)_3_(OH)_3_(H_2_O)_3_ (Figure 2a,b). TGA analyses show an additional weight loss of around 330 °C that is related to the decomposition of the added DHBA ligands (Supporting Information, Section S5). The above-discussed characterization is related to the average structure at the long scale, which is useful to assess the connectivity and porosity of the MOF materials. However, it is imperative to apply complementary structural characterization probes to assess the local structure of the Zr_6_O_8_ nodes upon modification with the catechol ligands. In this regard, FTIR spectroscopy could shed some light on the coordination of the DHBA ligands to the MOF-808 nodes. Thus, simulations of the whole IR spectrum of the chemically activated MOF-808-P would be helpful to fully understand the chemistry of the functionalized material. Our DFT calculations indicated that the theoretical IR spectrum of MOF-808-P is characterized by the presence of several characteristic bands within the range of 500–4000 cm^–1^. In particular, the experimental FTIR spectrum of MOF-808-P shows main bands located at 655, 1380, 1445, 1560, and 1620 cm^–1^ (Figure 3), in agreement with the previous work.^19,27,37−40^ However, a complete assignment of the experimental IR spectrum for MOF-808 has not been reported in the recent literature so far due to the difficulties to characterize all of the vibrational modes linked to the framework. Typically, partial assignments have been discussed for tracking the presence of certain functional groups, which is a useful strategy to flag postsynthetic modifications.^27,37−40^ The high accuracy of our theoretical calculations allows us to fill this gap by proceeding with the detailed assignment of the whole IR experimental spectrum of MOF-808-P. In Figure 3, the theoretical IR spectrum of MOF-808-P obtained from first-principles calculations is compared with the experimental data, showing a good agreement. For this task, we have used the methodology developed in our previous work^41^ and is explained in detail in Supporting Information, Section S11.

In detail, the four main IR signals within the 1300–1650 cm^–1^ range correspond to vibrations associated with the organic linkers of MOF-808-P. In particular, the band at 1620 cm^–1^ comes from the strong C=C stretching of aromatic rings, while all of the rest are due to the COO stretching modes. The signal located in 1560 cm^–1^ corresponds to the asymmetric COO stretching, while the bands at 1380 and 1445 cm^–1^ result from the COO symmetric stretching modes in combination with other ring deformations. The mixed nature of these vibrational modes (they are not pure COO stretching modes) is responsible for the observed splitting of two peaks. Moreover, in the theoretical calculation, we find further splitting of this band on several groups corresponding to very similar modes. This fact makes the full assignment of the experimental IR data of MOF-808 extremely challenging.

Interestingly, our calculations also corroborated that the group of weak signals centered at 1100 cm^–1^ seen for MOF-808 after chemical activation (MOF-808-P) is attributed to collective vibrations of the hydroxo/water ligands within the node—that is, to the vacancy sites within the Zr_6_O_8_ node in MOF-808. The visualization of the vibrational modes of MOF-808 in that region clearly supports this point. We hypothesize that this IR signal can be used as a fingerprint of the chemical activation of the MOF-808 nodes since this broad signal is a consequence of the hydrogen bonds constituted among the hydroxo/water ligands. This effect cannot be reproduced in the theoretical spectrum due to the absence of a real distribution of O···H distances.

In the low energy region of the spectrum, an intense and broad signal centered at 655 cm^–1^ is observed (Figure 3). This feature can be assigned to collective vibrations of the Zr_6_O_8_ node within MOF-808 involving multiple Zr–oxo bonds. Moreover, most of these modes have a large spatial extension and are also coupled with the vibrational modes of the carboxylate groups of the linkers, thereby affecting as well the signals linked to the aromatic rings. It is worth noting that there is also a second band associated with similar Zr–oxo vibrations located slightly below 500 cm^–1^, which is usually not accessible in conventional IR experiments. The precise assignment of the IR fingerprint of the zirconia node might be very useful for monitoring a plethora of postsynthetic modifications that imply changes in the Zr–oxo bonds, including metalation reactions.^15^

**Figure 4:**
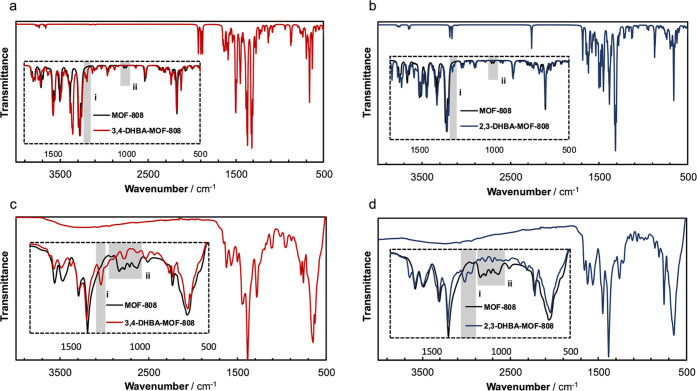
Comparison of DHBA- and pristine MOF-808 DFT-calculated (a and b) and experimental (c and d) IR spectra. i band: C–OH stretching vibrations of the DHBA ligands. ii bands: collective vibrations of the hydroxo/water ligands within the activated MOF-808 node, only present in the pristine material.

Next, the local structural changes occurring upon the modification of MOF-808 with DHBA ligands were studied in detail using FTIR spectroscopy. This is a very useful characterization approach to assess not only the incorporation of new ligands to the MOF-808 framework but also to elucidate their binding to the Zr–oxo nodes.^19,40^ The experimental IR spectra collected on MOF-808 after functionalization with 2,3- and 3,4-DHBA ligands are shown in Figure 4. After incorporation of the DHBA into the MOF-808, the appearance of a new band at ∼1250 cm^–1^ is observed for both ligands. To elucidate the nature of this vibrational mode, models of three representative configurations of 3-DHBA ligands bonded to the Zr–oxo node were computed (Supporting Information, Section S12). Our first-principles calculations confirmed that this new signal in the IR spectrum is linked to the C–OH stretching vibrations of the DHBA ligands. No significant changes with regard to the IR spectrum are observed for the different configurations (Supporting Information, Section S13). It is worth highlighting that the characterization of the incorporated DHBA ligands within MOF-808 by IR spectroscopy is particularly challenging since their structure resembles that of the BTC linker that acts as an MOF linker. This fact explains that most of the vibrational modes of the DHBA ligands overlap with those of the MOF-808 framework. Therefore, using our approach, the appearance of the signal linked to the C–OH stretching combined with the absence of the C=O stretching assigned to the free carboxylic group demonstrates the actual binding of DBHA to the zirconia nodes in MOF-808 through the carboxylate (Supporting Information, Section S12).

**Figure 5:**
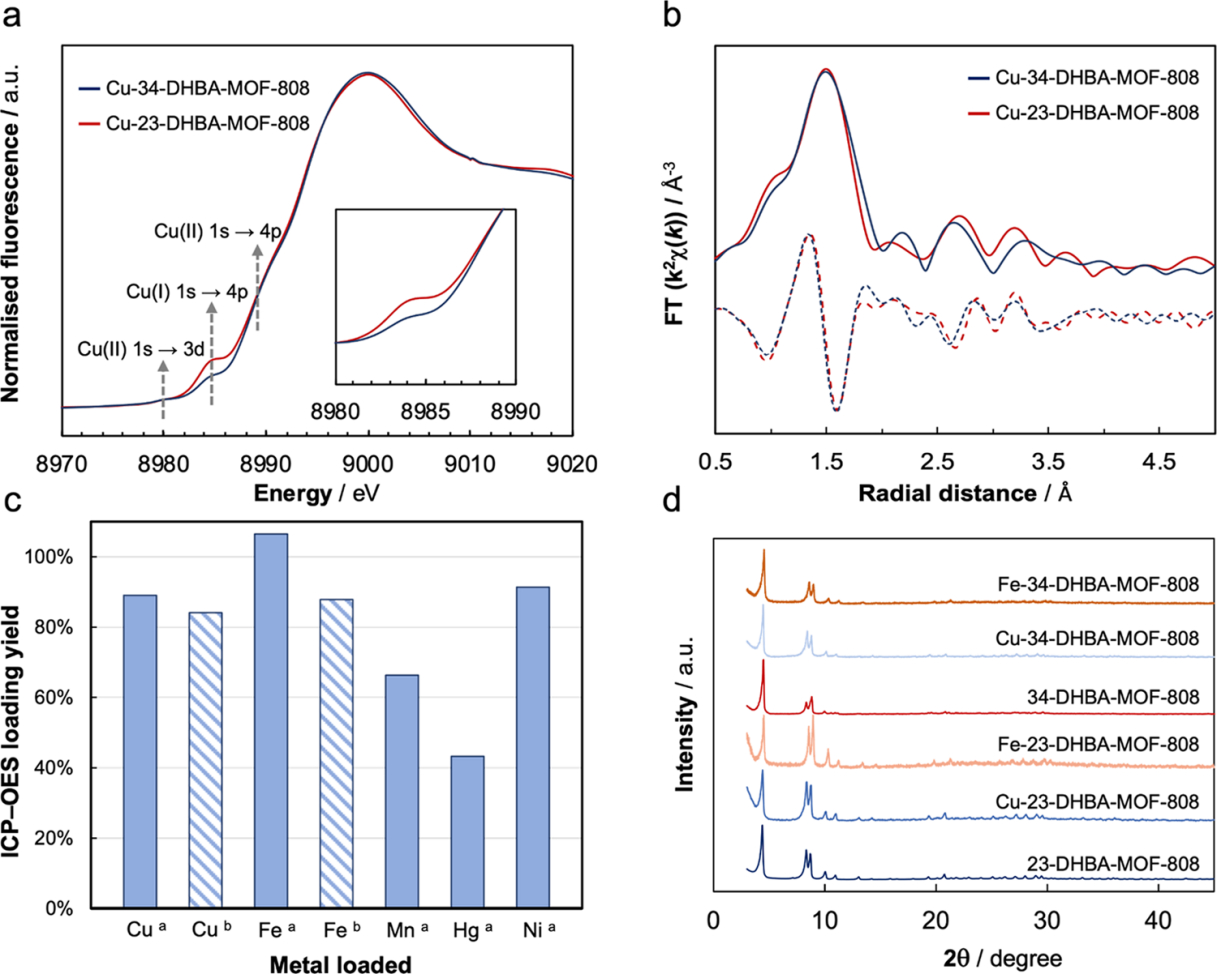
Normalized Cu-XANES spectra (a) and *k*^2^-weighted Cu-EXAFS data of Cu–2,3- and Cu–3,4-DHBA–MOF-808 magnitude (solid) and real component (dashed) (b). ICP-OES results of the loading efficiency test for d-metal loaded on 3,4-DHBA–MOF-808 (a) and 2,3-DHBA–MOF-808 (b,c).PXRD of Fe- and Cu–DHBA–MOF-808 systems (d).

The presence of a catechol moiety within the DHBA–MOF-808 systems allows further functionalization of the organic component through metalation reactions. Thus, the DHBA–MOF-808 materials were treated with triethylamine (3 equiv) in MeOH and subsequently with CuBr (3 equiv) at room temperature for 30 min. Using this procedure, the DHBA ligands incorporated in MOF-808 are deprotonated and metalated with copper, as determined by inductively coupled plasma-atomic emission spectroscopy (ICP-OES) analyses. The average symmetry and porosity of the MOF-808 framework are retained after metalation, as demonstrated by PXRD analyses, and BET surfaces of 1060 and 1090 cm^2^ g^–1^ were obtained for the 2,3-DHBA and the 3,4-DHBA–MOF-808, respectively (Figure 5d). Field emission scanning electron microscopy (FE-SEM) images were collected to study the morphology of the samples, showing the presence of octahedral crystals characteristic of the MOF-808 with an edge size of 300 nm (Supporting Information, Section S6). Interestingly, similar metalation protocols were carried out to incorporate a variety of d-metals (nickel, cobalt, manganese, iron, mercury) in oxidation state +2 (Figure 5c; Supporting Information, Section S7) into the DHBA–MOF-808, demonstrating the versatility of this strategy. Metalation reactions do not undergo in the absence of a base able to deprotonate the hydroxo groups of the DHBA ligands. It is worth highlighting that the crystallinity of the DHBA–MOF-808 materials is not compromised under basic conditions, as seen by PXRD analyses, in contrast to MOF-808 (Figure 5d).^42^

Cu K-edge X-ray absorption near-edge structure (XANES) and extended X-ray absorption fine structure (EXAFS) experiments were performed to assess the single-site nature of the copper sites. The appearance of a pre-edge feature at 8983 eV related to the 1s→4p transition shows the presence of Cu(I) in the precatalytic species (Figure 5a).^43^ The presence of Cu(I) within DBHA–MOF-808 is expected since the reduction potential of the DHBA ligand is typically high enough to stabilize many d-block metals in low oxidation states.^34^ Additional pre-edge features of Cu(II) were appreciated: 1s → 3d transition at 8978 eV and 1s → 4p transition at 8989 eV, showing the presence of this oxidation state in the samples.^44,45^ Interestingly, the difference in the relative intensity of the pre-edge signals for both materials could indicate that the Cu(I)/Cu(II) ratio is higher in 2,3-DHBA–MOF-808 compared to that of its 3,4-DHBA analogue. The different oxidation rates of copper within the isomers suggest a different behavior of the metal depending on the orientation of the catechol moieties toward the pores. Furthermore, the two Cu–DHBA–MOF-808 samples show almost identical features in the EXAFS data, demonstrating the occurrence of similar local structures (Figure 5b). In particular, the main peak centered at ∼1.5 Å (without phase correction) can be assigned to the Cu–O bond expected from the Cu–catecholate, proving the single nature of the copper sites.

Pair distribution function (PDF) analyses based on synchrotron total X-ray scattering data were performed on different metalated 2,3- and 3,4-DHBA–MOF-808 materials (Supporting Information, Section S8). The PDF technique is a powerful method to elucidate not only the local structure of the metal sites bonded to the catechol groups but also to ascertain the lack of metal oxide nanoparticles formed as byproducts. Differential analysis of the PDF data (d-PDF) has the potential of highlighting new atom–atom distances following metalation of the catechol moieties. d-PDF data were obtained in real space by subtracting the PDF of the bare DHBA–MOF-808 from that of the metalated material after applying a normalization factor. The d-PDF data obtained for the Cu–DHBA–MOF-808 materials show main signals at ∼2.01 and ∼3.34 Å linked to the copper centers, in agreement with the presence of single sites attached to the zirconia nodes. In addition, the lack of d-PDF peaks at the medium-range scale corroborates the absence of parasite metal oxide nanoparticles. This evidence demonstrates the versatility of DHBA–MOF-808 systems to stabilize single metal sites, opening new possibilities for designing catalytic materials.

**Figure 6:**
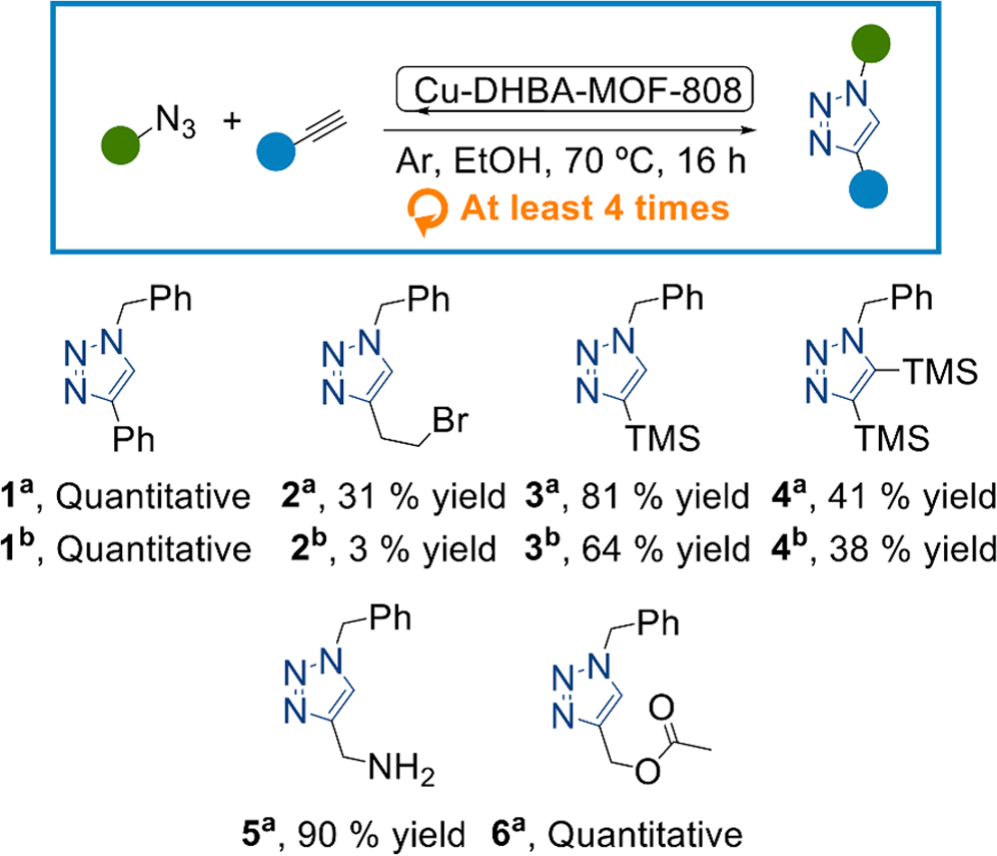
Cu(I)-catalyzed triazole formation from an alkyne and an azide. Cu–DHBA–MOF-808 catalyst (1 mol %), azide (0.8 mmol), and alkyne (0.9 mmol). Different catalysts were used: ^a^ Cu–2,3-DHBA–MOF-808 and ^b^ Cu–3,4-DHBA–MOF-808.

As a proof of concept, the accessibility of the single copper sites within the DHBA–MOF-808 materials for catalytic transformation was assessed for the archetypal 1,3-dipolar cycloaddition between an azide and an alkyne. The reaction proceeds in good yields for both materials, with 1,4 regioselectivity in the formation of the triazine ring. This behavior can be explained by the presence of Cu(I) species under catalytic conditions. As discussed before, we hypothesize that Cu(I) is stabilized within the DHBA–MOF-808 structure due to the redox activity of the catechol group, thereby avoiding its rapid oxidation to Cu(II) in ethanol. This strategy could also be used for the stabilization of other metals such as Fe, Ni, or Mn in a low oxidation state. To identify differences in the catalytic activity for the two isomers of DHBA–MOF-808, we performed a scope of different alkynes using benzyl azide as the coupling partner. Interestingly, Cu–2,3-DHBA–MOF-808 showed a higher yield for a broad scope of substrates compared to its regioisomer, as expected for the higher amount of Cu(I). It is worth highlighting the versatility of the material, which can catalyze the coupling of both terminal and intern alkynes and a variety of different functional groups such as nucleophilic/basic (**5**), electrophilic (**2**), hydrolyzable (**6**), and bulky substituents (**3** and **4**) (Figure 6). Recyclability tests were performed using the model reaction between benzyl azide and phenylacetylene. The reaction was repeated up to 4 times for product **1**, retaining good yields over the different cycles and MOF long-range order after catalysis (Supporting Information, Section S10).

## Conclusions

In this work, we have simulated and assigned the IR spectrum of pristine and activated MOF-808 as an approach to characterize the presence of vacancy sites within the Zr_6_O_8_ nodes. We tracked the binding of catechol–benzoate ligands to MOF-808 clusters by the disappearance of a group of characteristic IR signals centered at *ca.* 1100 cm^–1^. Based on our calculations, we could elucidate that these bands are associated with complex vibrational modes of the labile aquo–hydroxo ligands, located in the vacancy sites within the equatorial plane of the Zr_6_O_8_ nodes. We further functionalized MOF-808 upon binding of d-metals to the catechol moieties for catalysis. The single nature of the added metal sites has been thoroughly assessed by synchrotron XAS and PDF techniques. In the case of copper, we demonstrated that the material is active in 1,3-dipolar cycloaddition. We believe that the synergistic strategy shown in this work by combining computational-assisted IR interpretation of experimental data could be expanded to harness the chemical modifications of the Zr_6_O_8_ nodes in other related materials beyond MOF-808.

## Supporting Information Available

Contents of the material supplied including characterization data (PXRD, FTIR, nitrogen adsorption experiments, TGA, SEM, PDF, NMR), and synthetic and computational details (PDF)
