# Supplementary material for: Revisiting Vibrational Spectroscopy to Tackle the Chemistry of Zr6O8 Metal-Organic Framework Nodes
Source: ACS Appl Mater Interfaces. 2022 May 31;14(23):27040–7. doi: 10.1021/acsami.2c04712 (PMC9204696; doi:10.1021/acsami.2c04712)
Supplement: Supplementary file 1 — am2c04712_si_001.pdf [file am2c04712_si_001.pdf]

Supporting Information: Revisiting Vibrational Spectroscopy to Tackle the Chemistry of Zr_6O_8 Metal-Organic Framework Nodes

Ignacio Romero-Muñiz,[†] Carlos Romero-Muñiz,[‡] Isabel del Castillo-Velilla,[†] Carlo Marini,[¶] Sofía Calero,[§] Félix Zamora,[*,†,‖,⊥] and Ana E. Platero-Prats[*,†,‖,⊥]

†*Departamento de Química Inorgánica Facultad de Ciencias, Universidad Autónoma de Madrid Campus de Cantoblanco, 28049 Madrid (Spain)*

‡*Departamento de Física Aplicada I, Universidad de Sevilla, E-41012, Seville (Spain)*

¶*CLAESS beamline, ALBA Synchrotron,08290 Cerdanyola del Vallès (Spain)*

§*Materials Simulation & Modelling, Department of Applied Physics Eindhoven University of Technology, 5600MB Eindhoven (The Netherlands)*

‖*Condensed Matter Physics Center (IFIMAC) Universidad Autónoma de Madrid Campus de Cantoblanco, 28049 Madrid (Spain)*

⊥*Instituto de Investigación Avanzada en Ciencias Químicas de la UAM, Universidad Autónoma de Madrid Campus de Cantoblanco, 28049 Madrid (Spain)*

E-mail: felix.zamora@uam.es; ana.platero@uam.es

Contents

S1 Synthesis **S-3**

S2 Powder X-ray diffraction **S-6**

S3 Fourier-transform infrared spectroscopy **S-14**

S4 Nitrogen adsorption isotherms **S-17**

S5 Thermogravimetric analyses **S-21**

S6 Scanning electron microscopy **S-24**

S7 Metalation studies **S-27**

S8 X-ray pair distribution function analyses **S-29**

S9 Nuclear magnetic resonance spectra **S-34**

S10 Catalytic tests **S-40**

S11 Computational details **S-43**

S12 Cluster models **S-46**

S13 Further IR theoretical spectra **S-49**

References **S-50**

S1 Synthesis

List of materials

- **MOF-808-F**. As-synthesized MOF-808. This material has 6 non-structural formates and none open metal sites.
- **MOF-808-P**. Activated MOF-808. This material has 12 open metal sites occupied by 6 water molecules and 6 hydroxo anions.
- **2,3-DHBA-MOF-808**. MOF-808 functionalized with 2,3-dihydroxobenzoic acid. It has 3 molecules of the DHBA ligand and 6 open metal sites occupied by 3 water molecules and 3 hydroxo anions.
- **3,4-DHBA-MOF-808**. MOF-808 fucntionalised with 3,4-dihydroxobenzoic acid. It has 3 molecules of the DHBA ligand and 6 open metal sites occupied by 3 water molecules and 3 hydroxo anions.
- **M-2,3-DHBA-MOF-808**. 2,3-DHBA-MOF-808 metalated with different metals.
- **M-3,4-DHBA-MOF-808**. 3,4-DHBA-MOF-808 metalated with different metals.

All other reagents were used as received from commercial suppliers unless otherwise stated. Reaction progress was monitored by **thin layer chromatography** (TLC) performed on aluminium plates coated with silica gel F254 with 0.2 mm thickness. Chromatograms were visualised by fluorescence quenching with UV light at 254 nm or by staining using potassium permanganate.

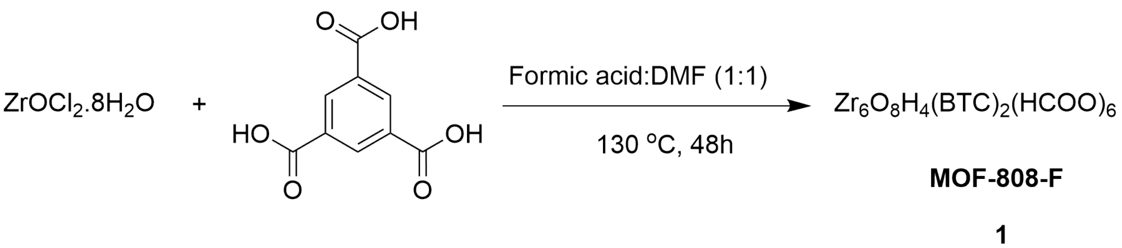

Scheme S1: Synthesis of MOF-808-F.

Synthesis of MOF-808-F (1).Benzene-1,3,5-tricarboxylic acid (210 mg, 1 eq, 1 mmol) and oxozirconium (IV) chloride (970 mg, 3.01 eq, 3.01 mmol) were dissolved in 90 mL DMF:formic acid (1:1). The reaction was heated at 130 °C for 48 hours in the oven. The white solid was washed three times with DMF, water and methanol. The resulting solid was dried at 60 °C for 1-2 h in the oven yielding **1** as a white powder. Yield: 1.4 g (35%) ^{1}H-NMR (300 MHz, DMSO-d_6) δ: 8.42 (s, 6H), 7.93 (s, 5H).

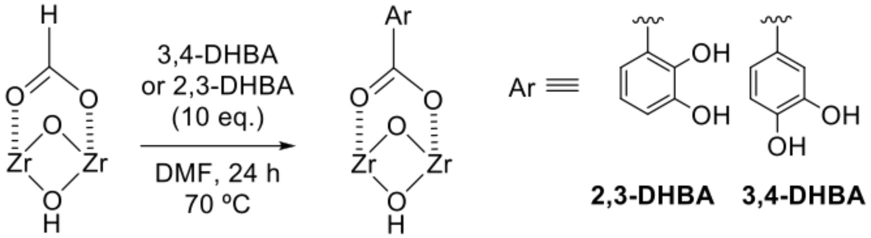

Scheme S2: functionalisation of MOF-808-F.

General procedure for (2) and (3). **1** (100 mg, 0.074 mmol, 0.1 eq.) was added to a stirred solution of the corresponding ligand (113 mg, 0.74 mmol, 1 eq.) in DMF (7.67 ml). The mixture was heated at 70 °C for 24 hours in a capped vial under stirring. The reaction mixture was centrifuged, and the white precipitate was washed three times with DMF, water and MeOH. The solid was dried *in vacuo* yielding **2** or **3** as white powders.

2,3-DHBA-MOF-808 (2) yield: 80 mg (76%) ^{1}H-NMR (300 MHz, DMSO-d6) δ: 8.61 (s, 3H), 8.08 (s, 1H), 7.26 (dd, J = 8.0, 1.6 Hz, 1H), 6.99 (dd, J = 7.8, 1.6 Hz, 1H), 6.74 (t, J = 7.9 Hz, 1H).

3,4-DHBA-MOF-808 (3) yield: 71 mg (65%) ^{1}H-NMR (300 MHz, DMSO-d6) δ: 8.96 (s, 3H), 8.51 (s, 1H), 7.77 (m, J = 8.0, 1.6 Hz, 2H), 7.22 (d, J = 7.8 Hz, 1H).

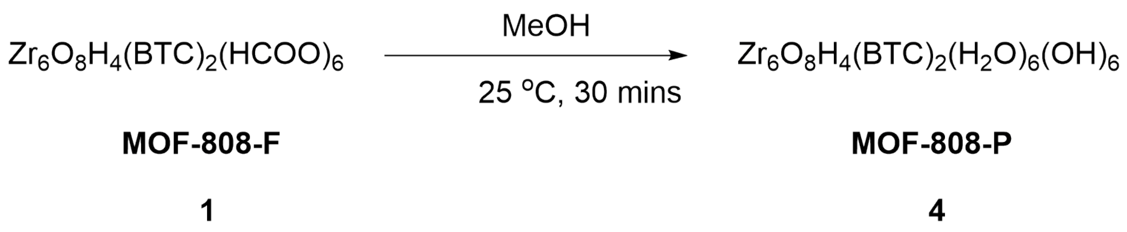

Scheme S3: Synthesis of MOF-808-P.

Chemical activation of MOF-808-F (4). MOF-808-P was prepared by washing MOF-808- F with MeOH at room temperature for 30 minutes. ^{1}H-NMR (300 MHz, DMSO-d$_6$) δ: 8.42 (s, 3H).

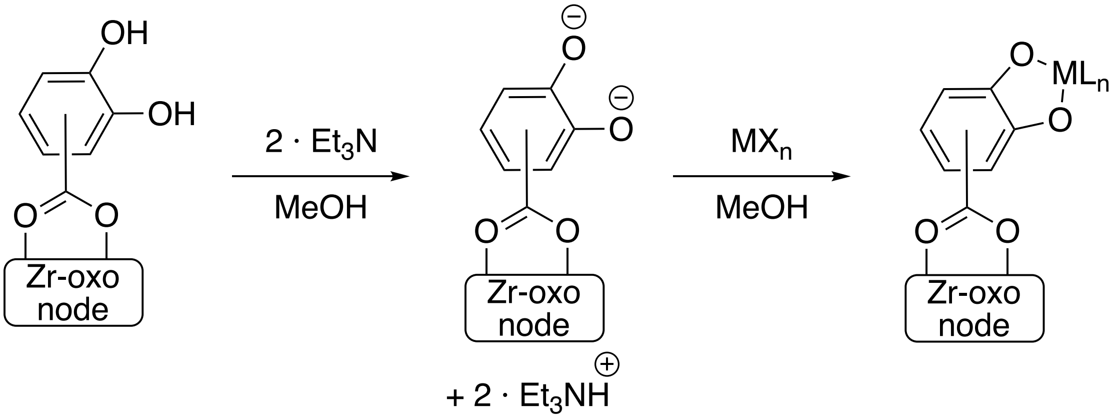

Scheme S4: Deprotonation and metallation reaction of DHBA-MOF-808.

Metalation of DHBA-MOF-808. DHBA-MOF-808 (25 mg, 1 Eq, 15 μmol) was added to a stirred solution of triethylamine (9.0 mg, 12 μL, 6 Eq, 89 μmol) in methanol (6.0 mL). The mixture was stirring during 15 minutes. Then, a metal salt (3 Eq, 44 μmol) was added. The reaction mixture was stirred at room temperature for 1 hour in a sealed vial. The solid was centrifuged and washed three times with methanol yielding the corresponding metalated MOF as a powder. Metal salts used: $FeCl_2$, $NiBr_2$, CuBr, $Mn(AcO)_2$, $Hg(AcO)_2$, $CoBr_2$.

S2 Powder X-ray diffraction

Powder X-ray diffraction (PXRD) data were measured with a Bruker D8 diffractometer with a copper source operated at 1600 W, with step size = 0.02 ° and exposure time = 0.5 s/step with a Bragg-Brentano geometry Samples were placed on a borosilicate sample holder. All the samples were grinded prior to analysis unless otherwise stated. Data were measured using a continuous 2θ scan from 3.0-45° θ. Le Bail refinement was made to all powder samples using JANA2006 program.[S1] Pseudo-Voigt peaks shapes were used along with a simple axial divergence correction. The lattice parameters, was refined against the values obtained from the CIF[S2] in the 2θ range of 3– 45°. The zero-point error was also refined.

Table S1: Le Bail refinement values for synthesized samples.

Table	Lattice constant / Å	R_{wp} / %	R_p / %
**MOF-808-F**	35.2793	11.27	5.13
**MOF-808-P**	34.3156	9.01	6.51
**2,3-DHBA-MOF-808**	35.3069	6.76	4.50
**3,4-DHBA-MOF-808**	35.2988	14.37	10.83
**Cu-2,3-DHBA-MOF-808**	35.2787	5.58	3.67
**Cu-3,4-DHBA-MOF-808**	35.2624	5.84	4.55

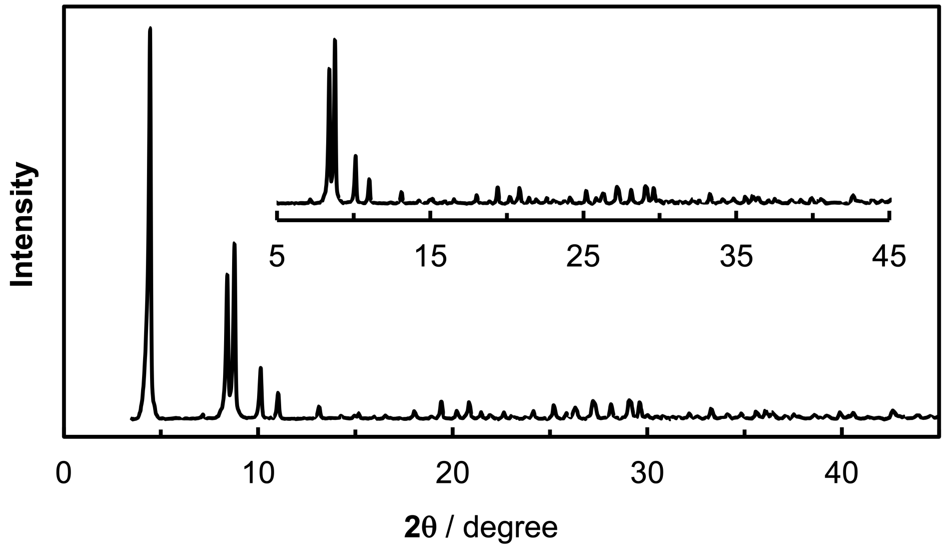

Figure S1: PXRD data of **MOF-808-F**.

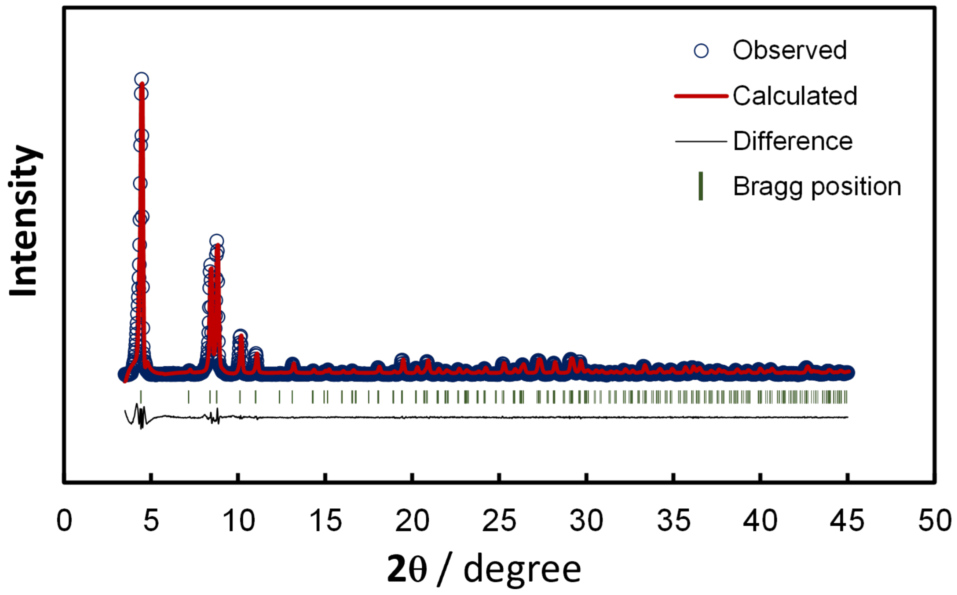

Figure S2: Le Bail fit of **MOF-808-F** PXRD data

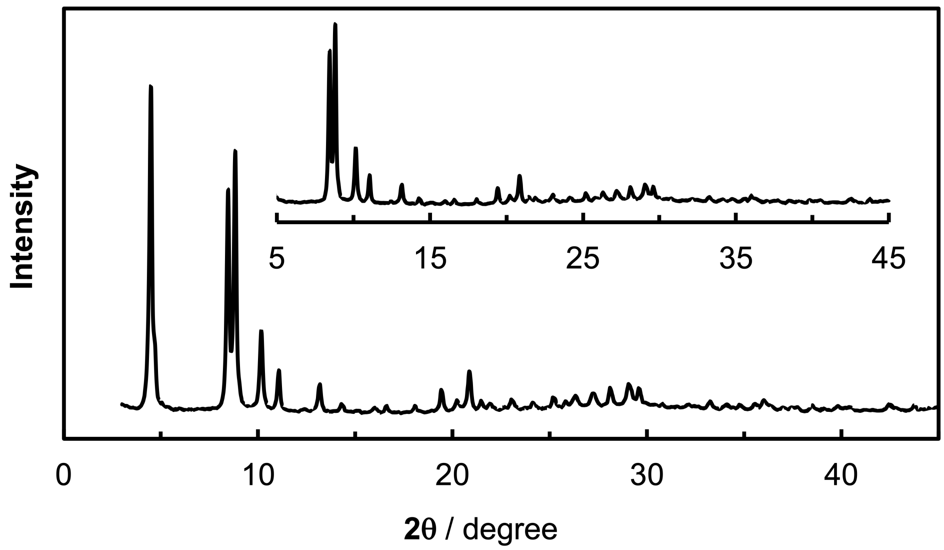

Figure S3: PXRD data of **MOF-808-P**.

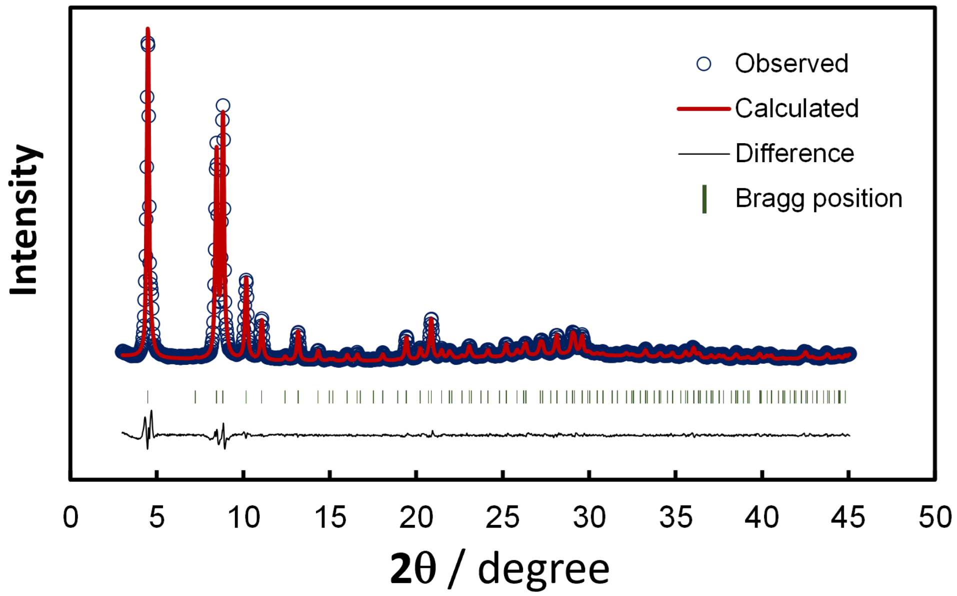

Figure S4: Le Bail fit of **MOF-808-P** PXRD data.

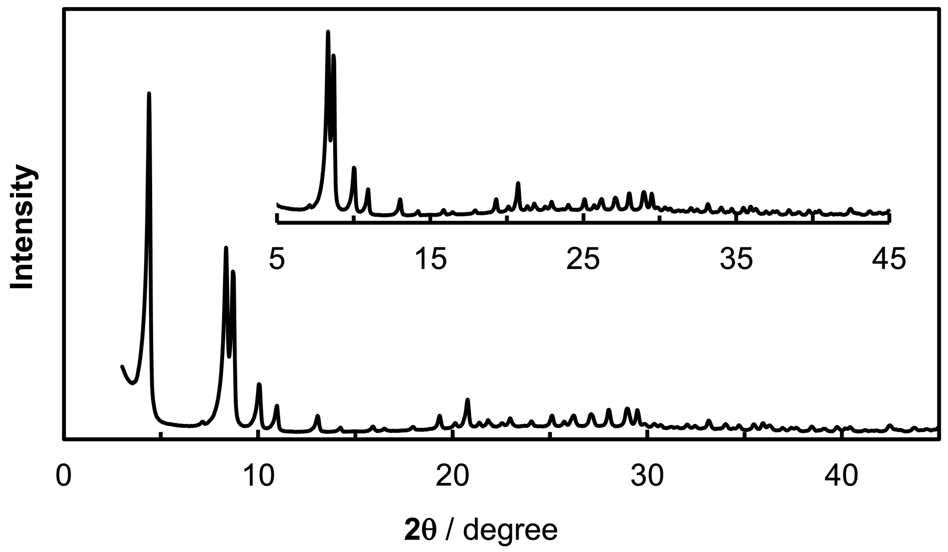

Figure S5: PXRD data of **2,3-DHBA-MOF-808**.

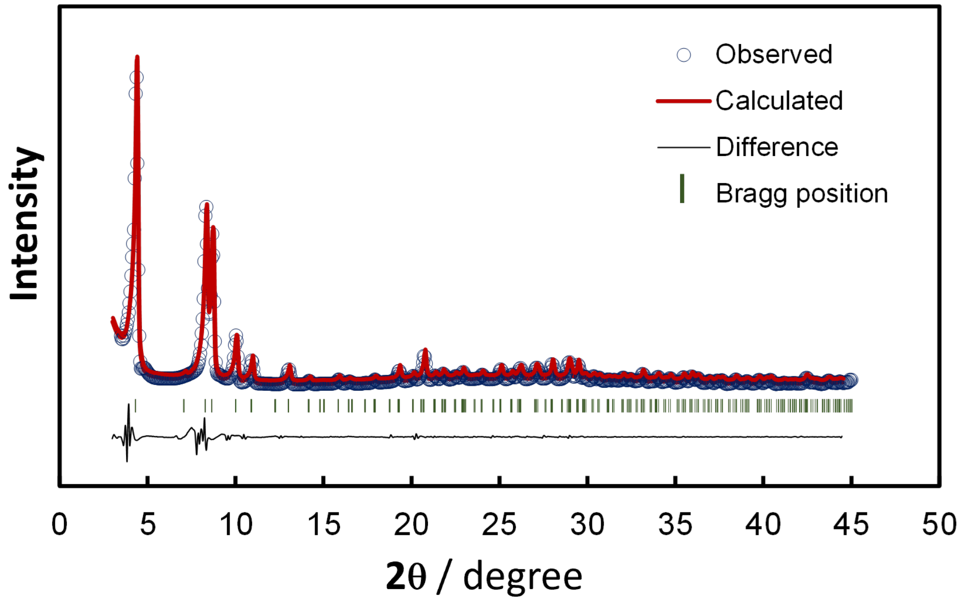

Figure S6: Le Bail fit of **2,3-DHBA-MOF-808 data** PXRD.

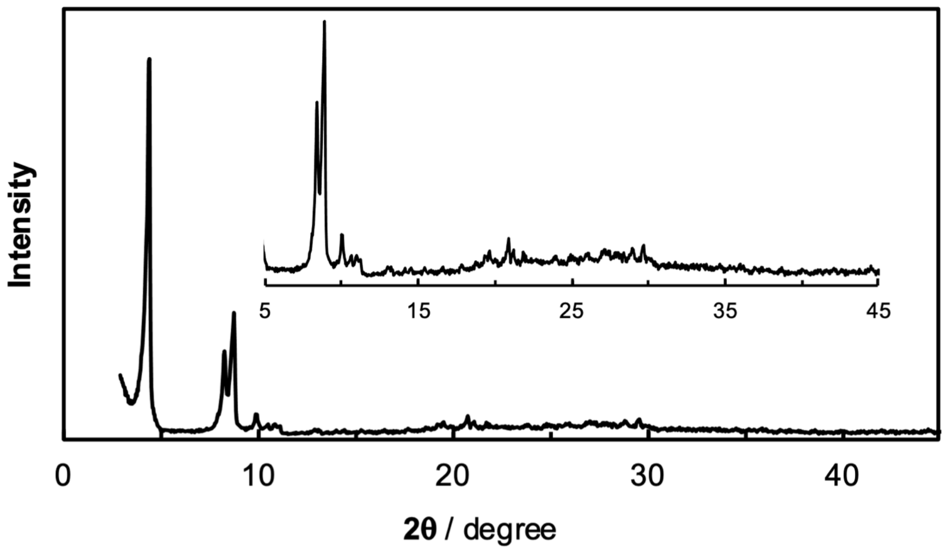

Figure S7: PXRD data of **3,4-DHBA-MOF-808**.

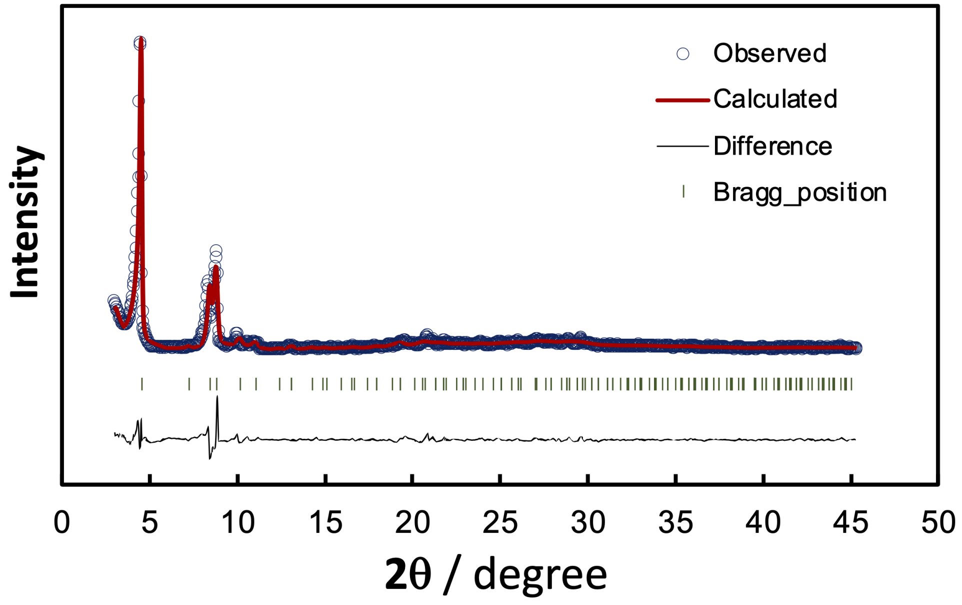

Figure S8: Le Bail fit of **3,4-DHBA-MOF-808 data** PXRD.

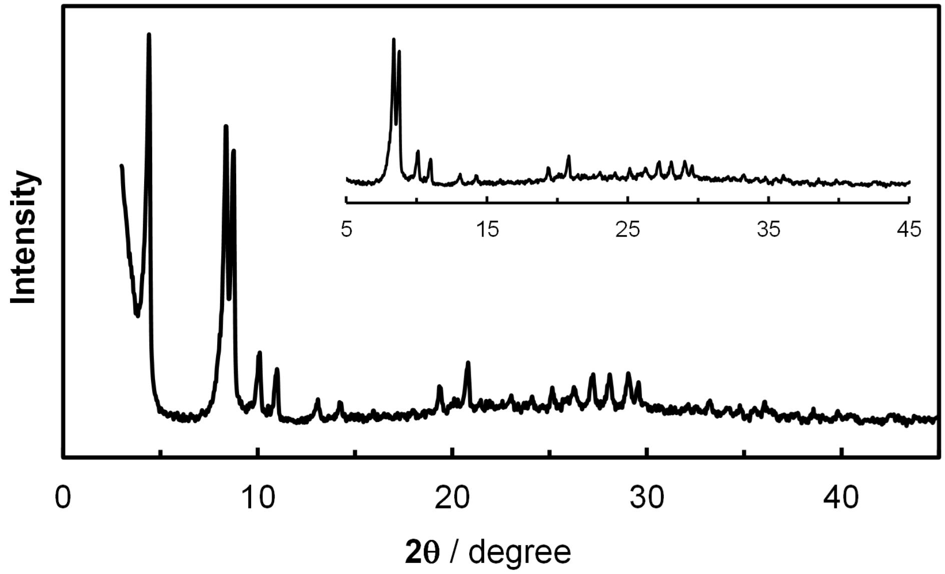

Figure S9: PXRD data of **Cu-23-DHBA-MOF-808**

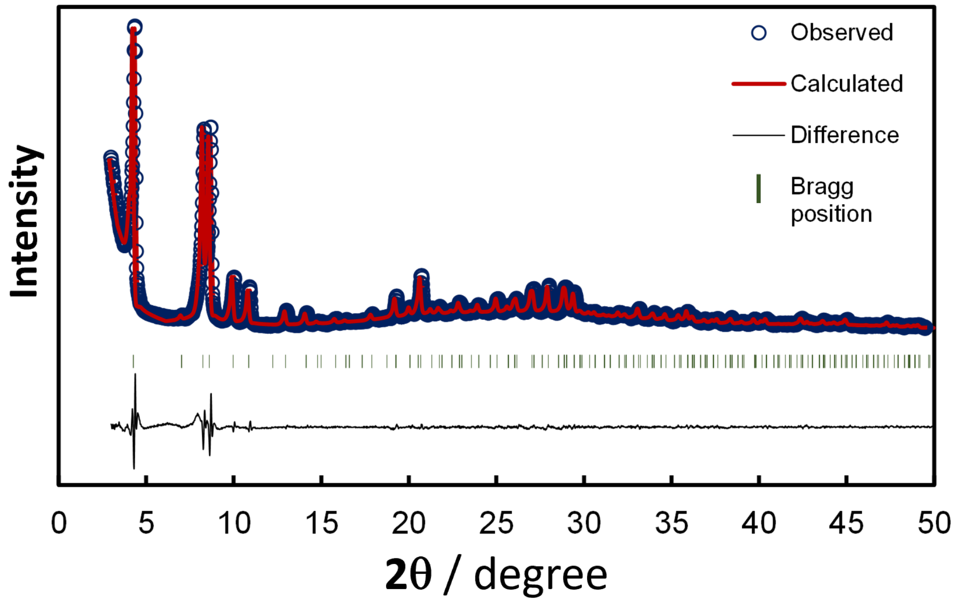

Figure S10: Le Bail fit of **Cu-2,3-DHBA-MOF-808** PXRD data.

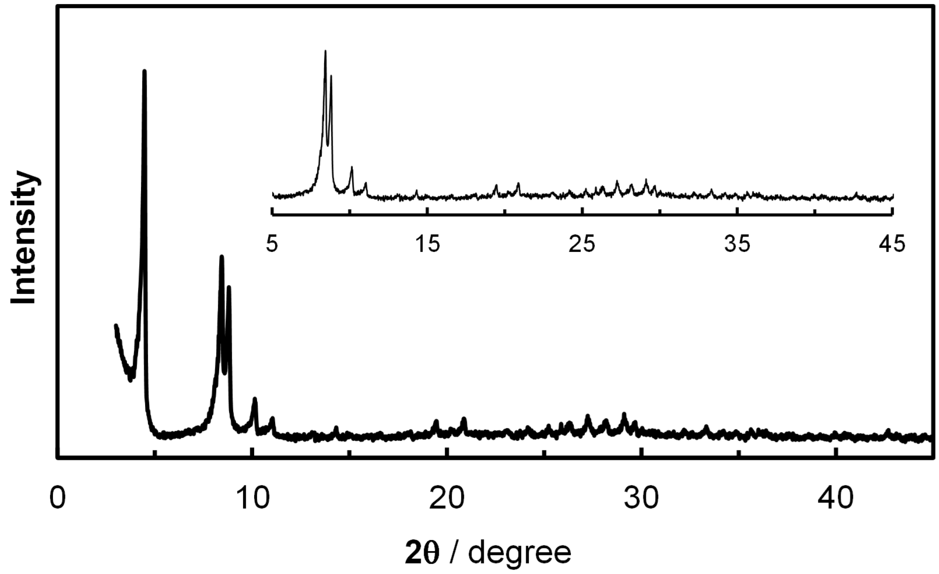

Figure S11: PXRD data of **Cu-3,4-DHBA-MOF-808**.

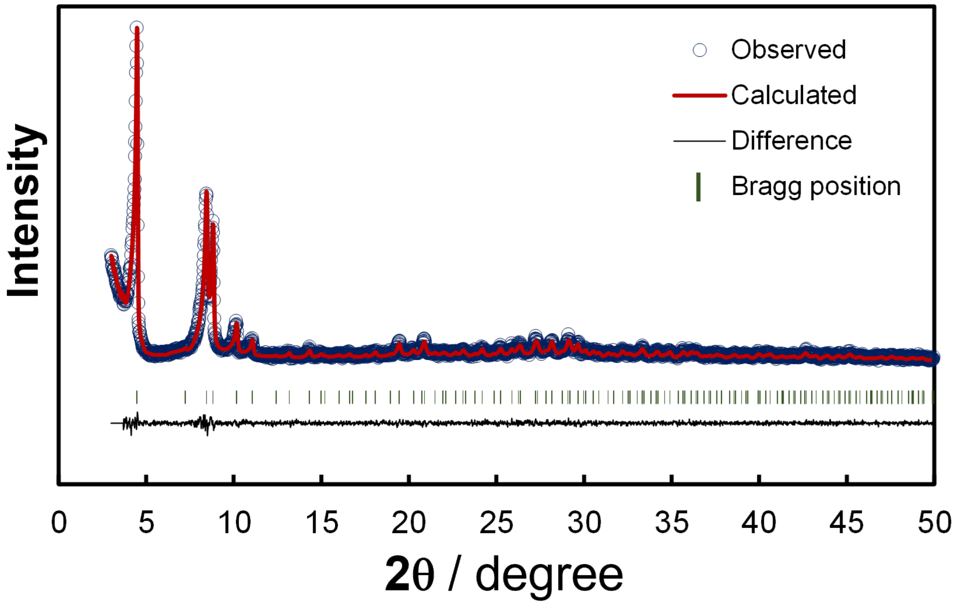

Figure S12: Le Bail fit of **Cu-3,4-DHBA-MOF-808** PXRD data.

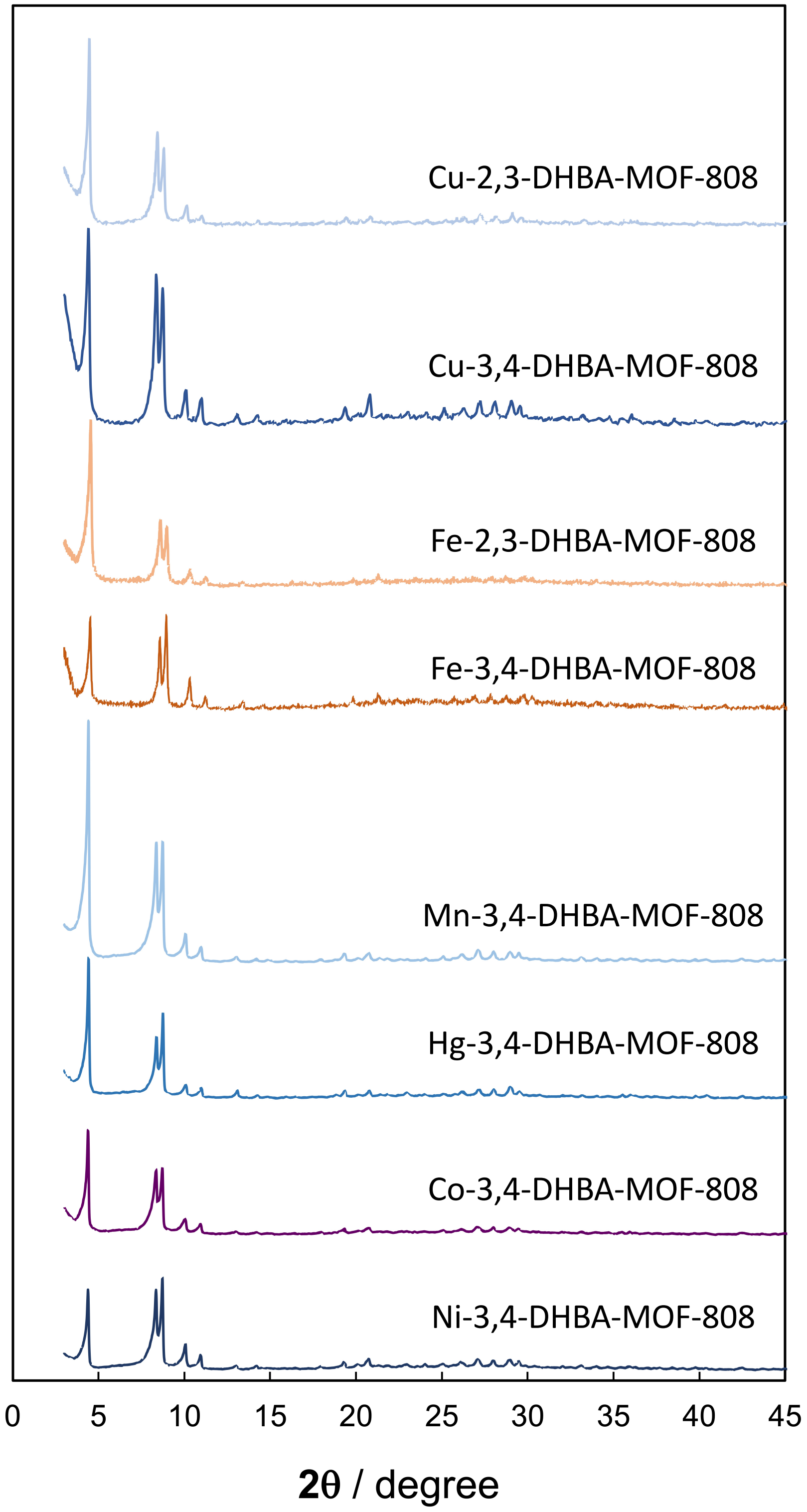

Figure S13: PXRD data of **Ni-3,4-DHBA-MOF-808**, **Co-3,4-DHBA-MOF-808**, **Hg-3,4-DHBA-MOF-808** and **Mn-3,4-DHBA-MOF-808**.

S3 Fourier-transform infrared spectroscopy

Attenuated total reflectance fourier-transform infrared spectroscopy (ATR-FTIR) spectra were recorded using FT-IR spectrometer Perkin Elmer model Spectrum 100, equipped with a diamond/ZnSe crystal. ATR-FTIR data was collected in the range of 500-4000 cm^{-1} with 32 scans per measure.

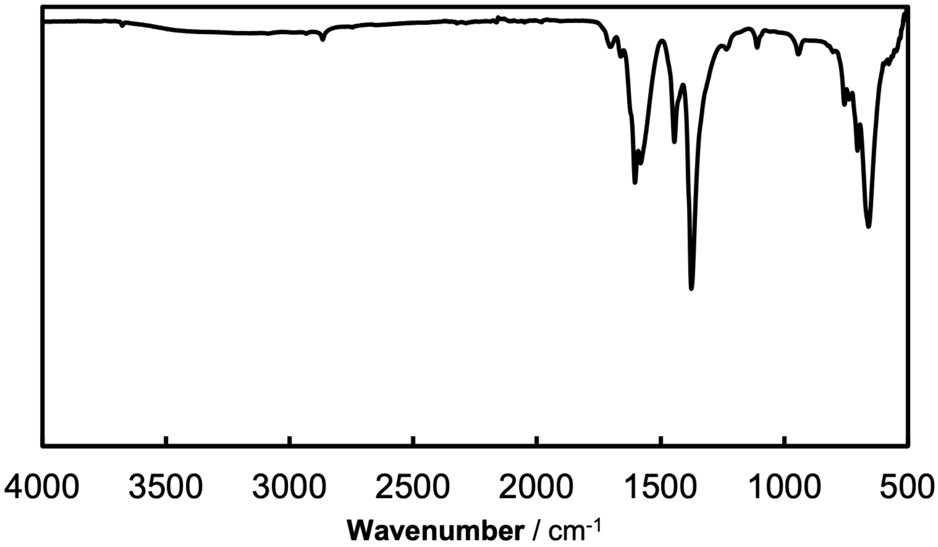

Figure S14: FT-IR spectrum of **MOF-808-F**.

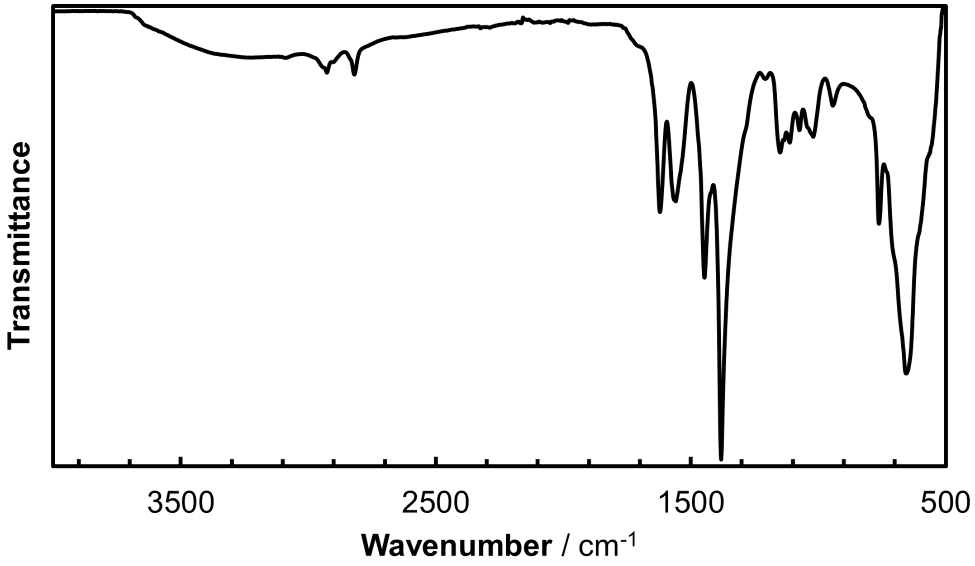

Figure S15: FT-IR spectrum of **MOF-808-P**.

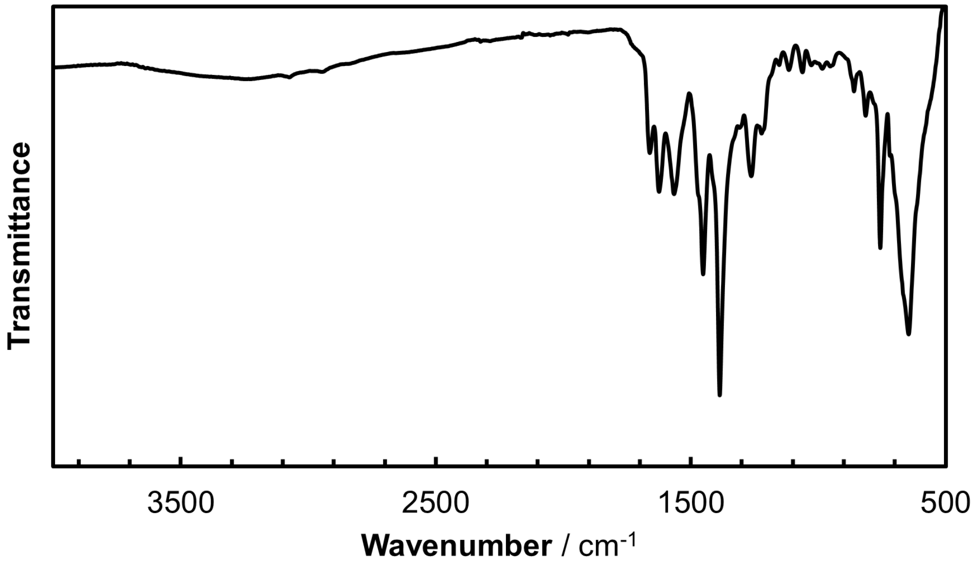

Figure S16: FT-IR spectrum of **2,3-DHBA-MOF-808**

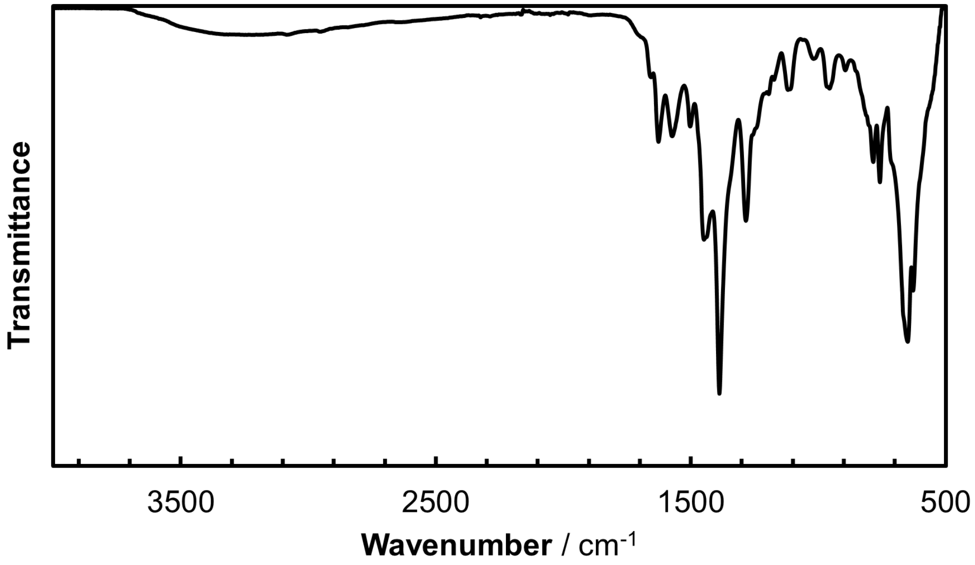

Figure S17: FT-IR spectrum of **3,4-DHBA-MOF-808**.

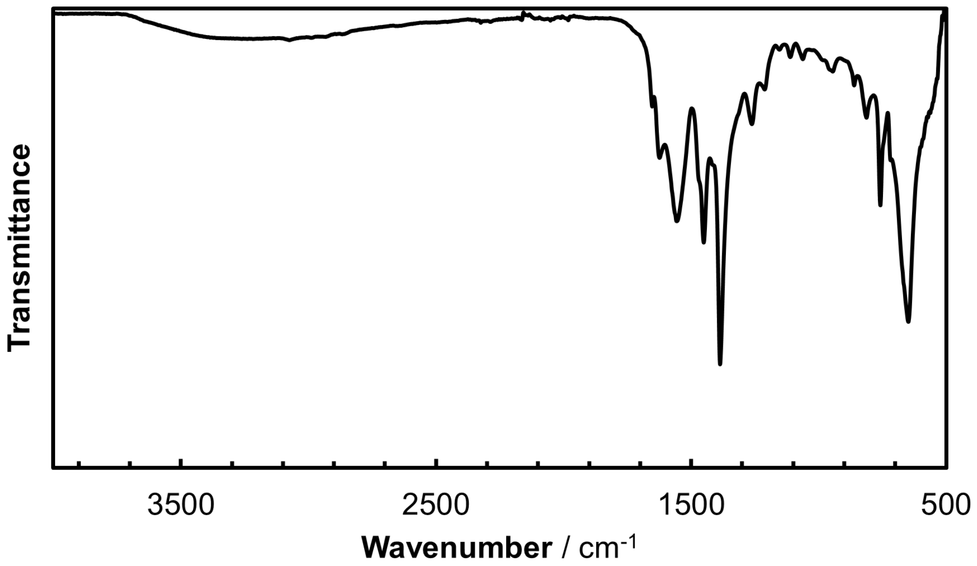

Figure S18: FT-IR spectrum of **Cu-2,3-DHBA-MOF-808**.

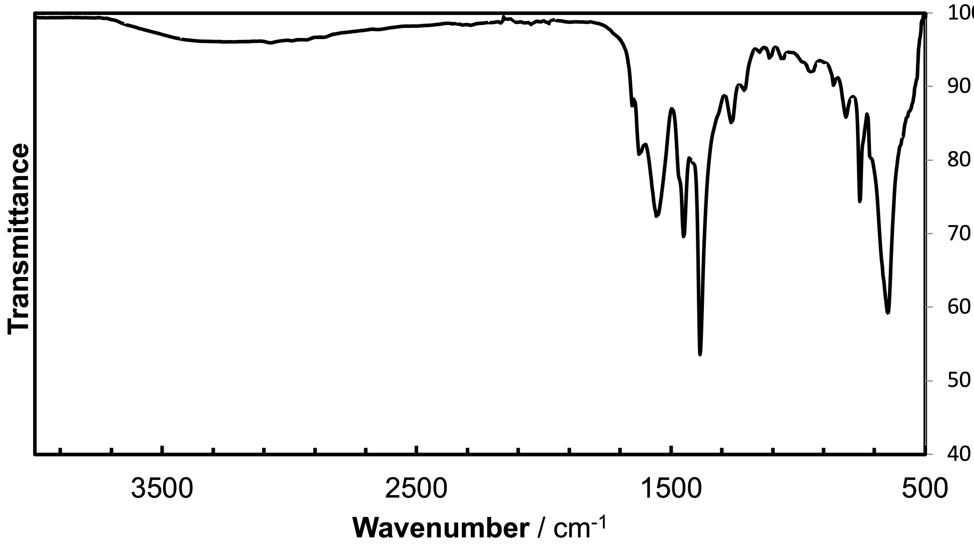

Figure S19: FT-IR spectrum of **Cu-3,4-DHBA-MOF-808**.

S4 Nitrogen adsorption isotherms

Nitrogen adsorption and desorption isotherms were measured at 77 K using a Micromeritics ASAP 2020 system. The samples were outgassed at indicated temperature for 16 h before the measurements. The specific surface areas (BET) were calculated by application of the Brunauer-Emmett-Teller equation taking the area of the nitrogen molecule as 0.162 nm^2. The linear range of the BET equation normally located between 0.05–0.35 P/P_0, was much narrower and displaced to lower relative pressures for all materials studied due to their microporous natures taking the linear range: P/P_0= 0.015–0.1. The micropore volume and external surface area, *i.e.* the area not associated with the micropores, were calculated using a t-plot analysis. taking the thickness of an adsorbed layer of nitrogen as 0.354 nm and assuming that the arrangement of nitrogen molecules in the film was hexagonal close packed. The mesopore volumes of the materials were calculated from the volume of gas adsorbed at a relative pressure of 0.6 on the desorption branch of the isotherms, equivalent to the filling of all pores below 50 nm, minus the microporosity calculated from the corresponding t-plot. The total pore volume was calculated from the volume of gas adsorbed at a relative pressure of 0.95 on the absorption branch of the isotherms.

Pore-size-distribution (PSD) curves were obtained from the adsorption branches using non-local density functional theory (NLDFT) method for a cylinder pore in pillared clays.

Table S2: Comparison of materials textural properties.

Material	Activation / °C	BET area / m^2g^{-1}	Pore width / Å	Micropore (total pore) vol. / cm^3
**MOF-808-F**	150	1375	18	0.47(0.54)
**MOF-808-P**	150	1712	18	0.66 (0.91)
**2,3-DHBA-MOF-808**	100	1225	15	0.44 (0.60)
**3,4-DHBA-MOF-808**	100	1126	15	0.41 (0.56)
**Cu-2,3-DHBA-MOF-808**	100	1060	15-11	0.37 (0.41)
**Cu-3,4-DHBA-MOF-808**	100	1090	15-11	0.39 (0.50)

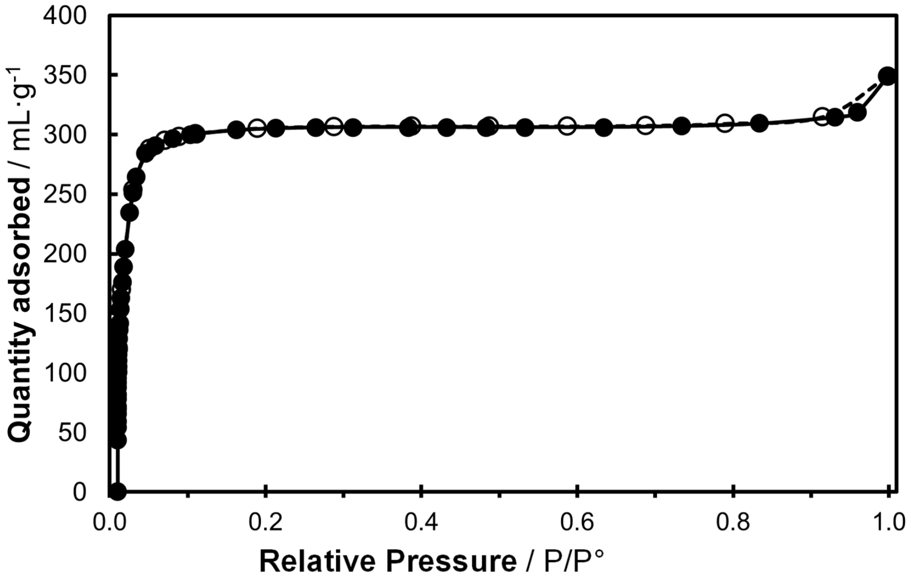

Figure S20: N_2 adsorption isotherm at 77 K of **MOF-808-F**.

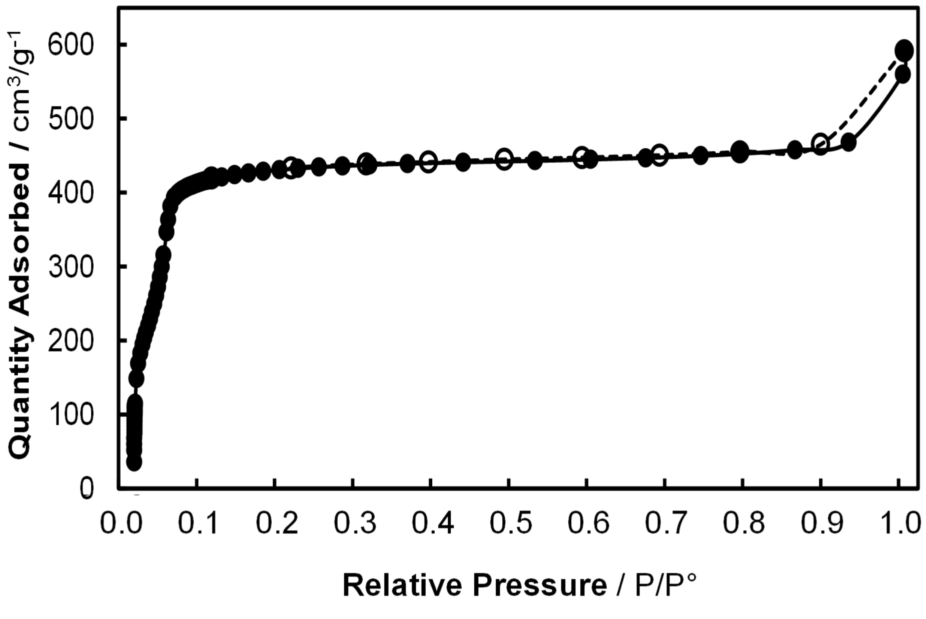

Figure S21: N_2 adsorption isotherm at 77 K of **MOF-808-P**.

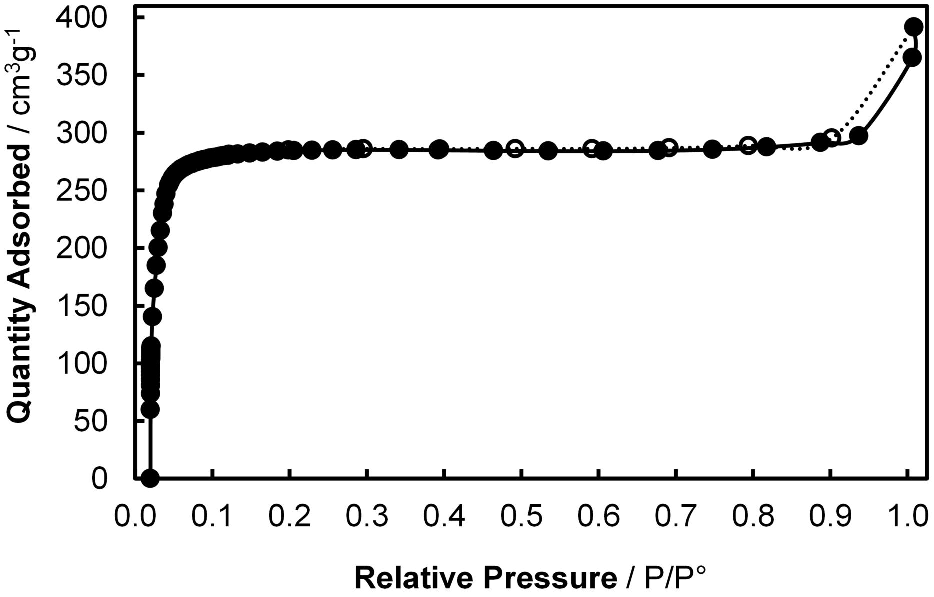

Figure S22: N_2 adsorption isotherm at 77 K of **2,3-DHBA-MOF-808**.

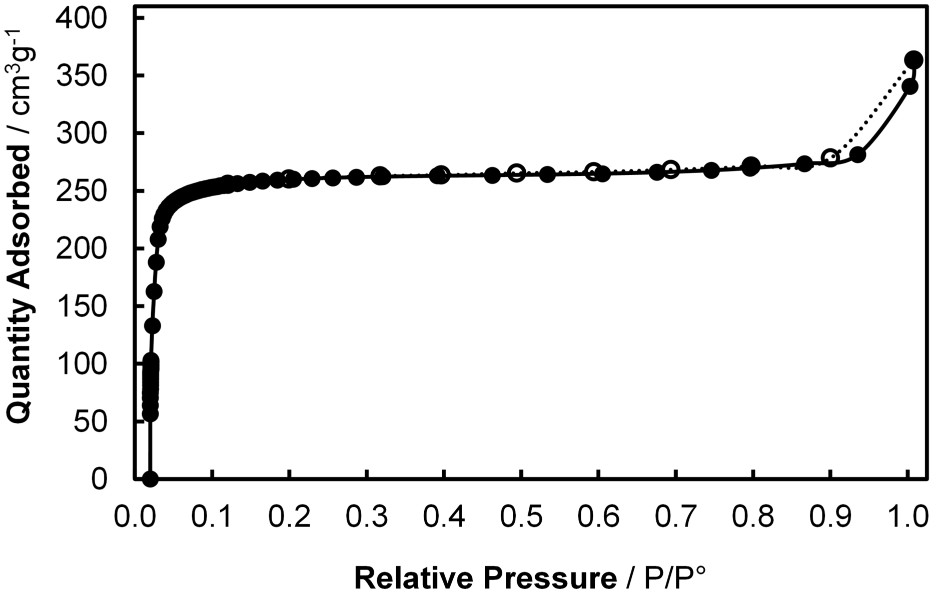

Figure S23: N_2 adsorption isotherm at 77 K of **3,4-DHBA-MOF-808**.

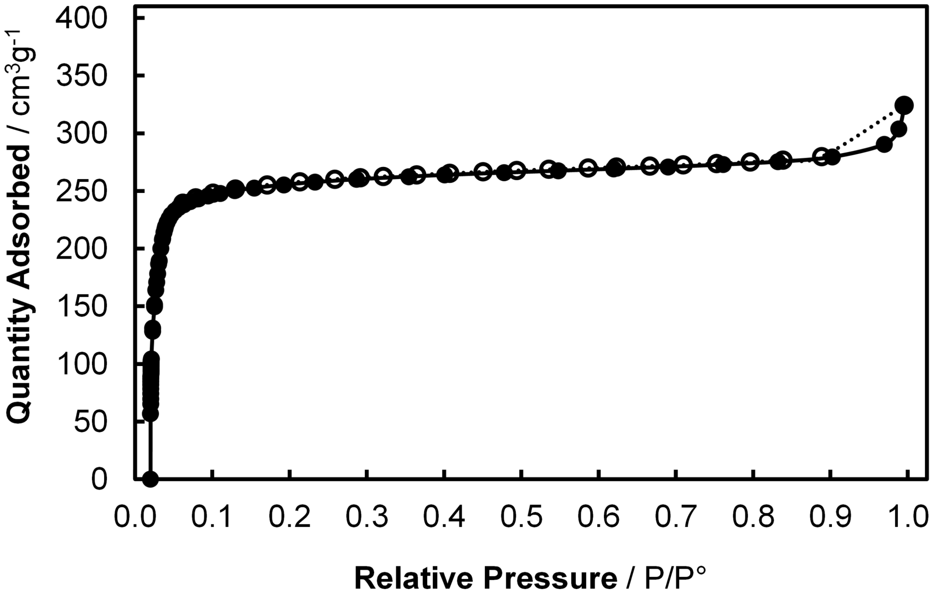

Figure S24: N_2 adsorption isotherm at 77 K of **Cu-2,3-DHBA-MOF-808**.

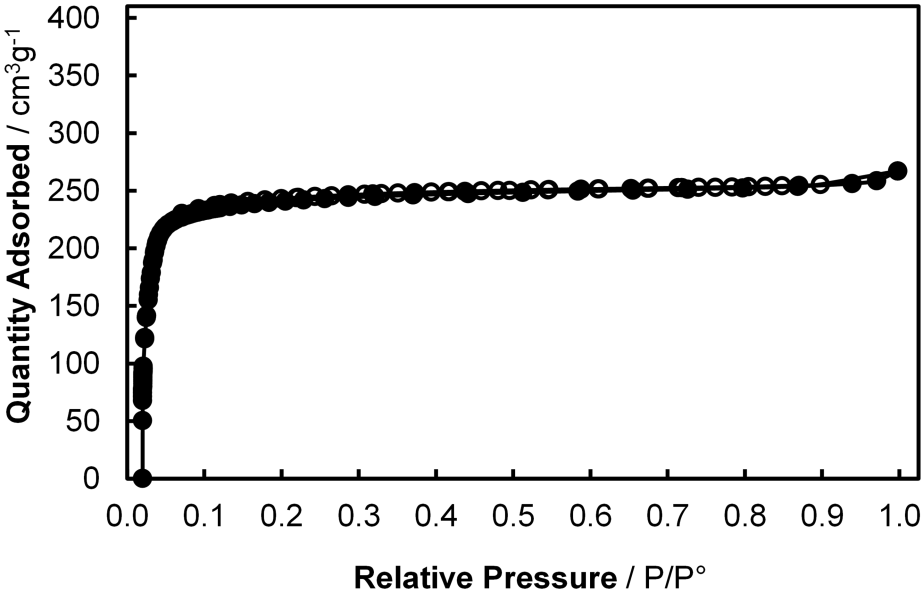

Figure S25: N_2 adsorption isotherm at 77 K of **Cu-3,4-DHBA-MOF-808**.

S5 Thermogravimetric analyses

Thermogravimetric analyses (TGA) were carried out on a TA Instruments Q500 thermobalance oven with a Pt sample holder and mass detector; N_2 was used as purge gas, at a flow rate of 90 mL/min; the samples were heated from 25 to 1000 °C at a rate of 10 °C/min

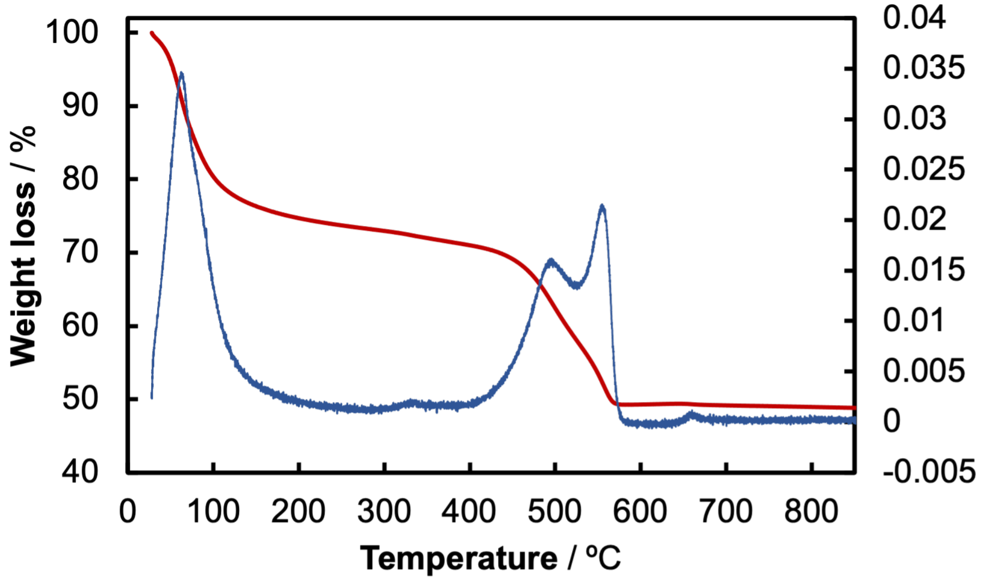

Figure S26: TGA analysis of **MOF-808-P** (red) and differential curve (blue).

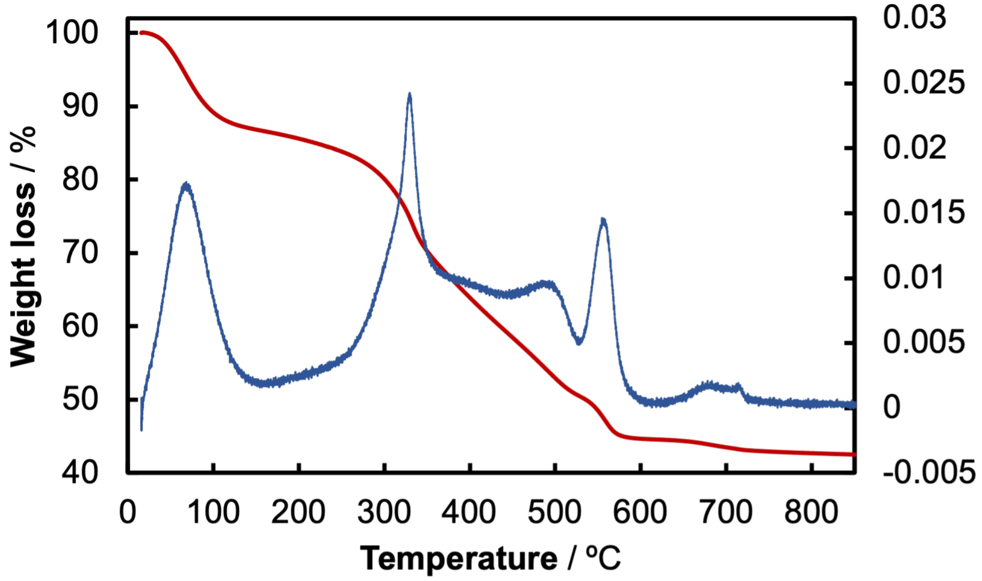

Figure S27: TGA analysis of **23-DHBA-MOF-808** (red) and differential curve (blue).

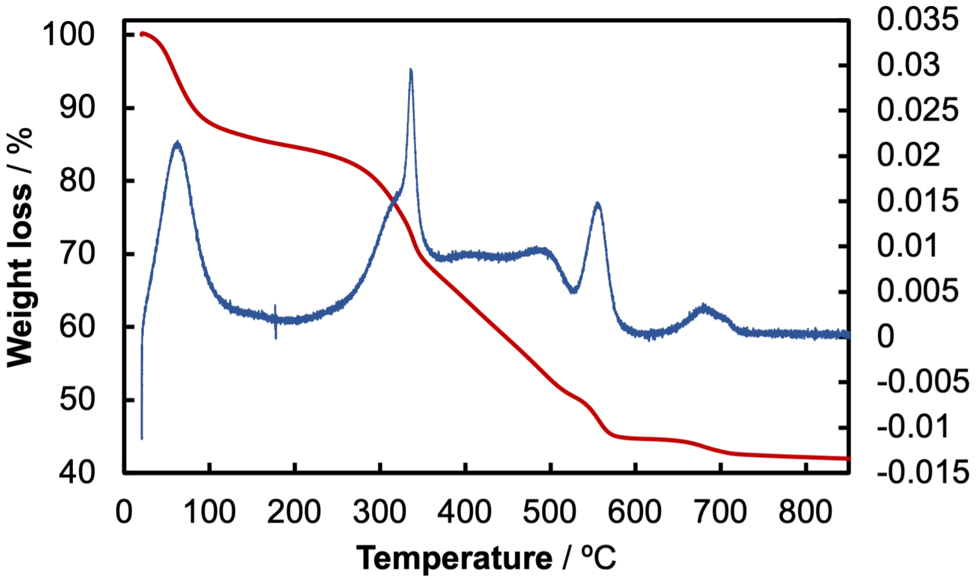

Figure S28: TGA analysis of **23-DHBA-MOF-808** (red) and differential curve (blue).

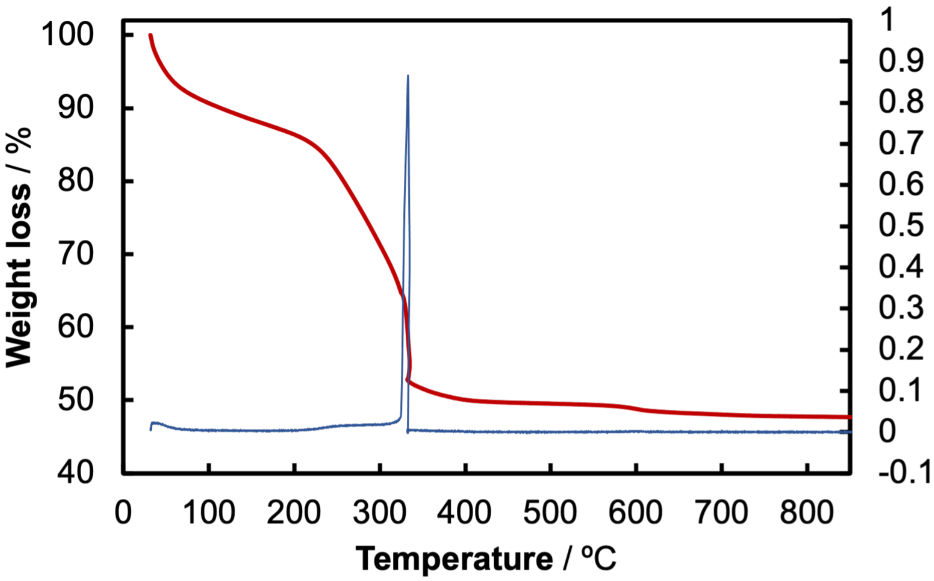

Figure S29: TGA analysis of **Cu-23-DHBA-MOF-808** (red) and differential curve (blue).

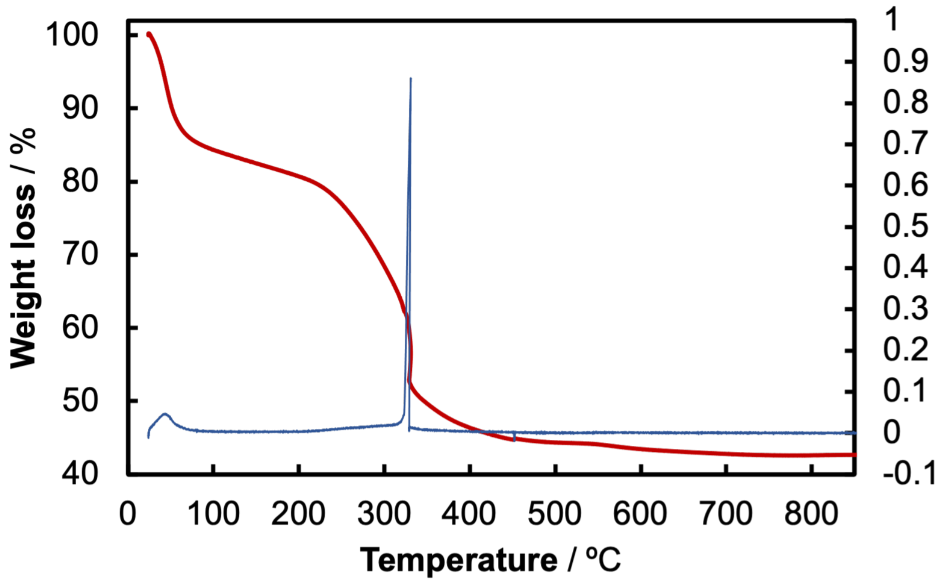

Figure S30: TGA analysis of **Cu-34-DHBA-MOF-808** (red) and differential curve (blue)

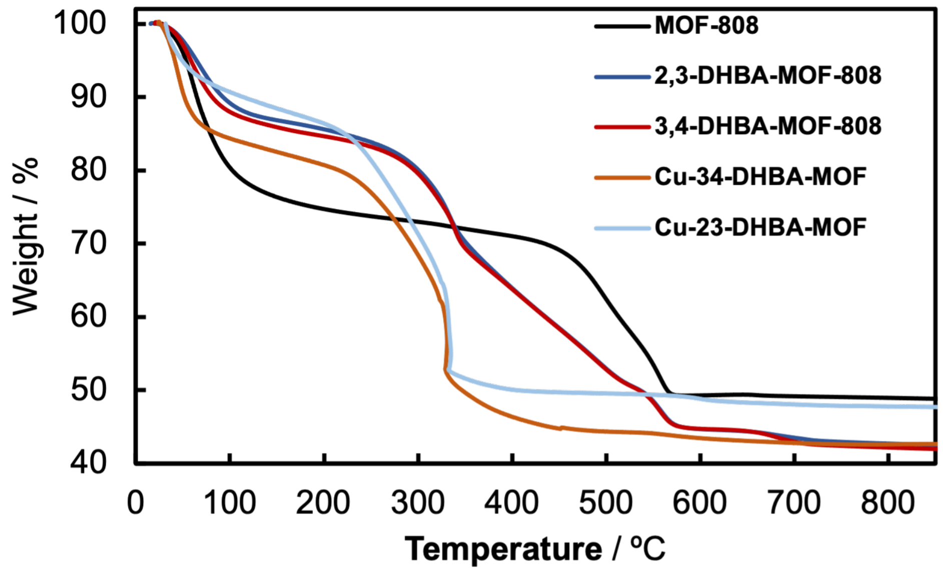

Figure S31: Comparison of TGAs MOFs materials.

S6 Scanning electron microscopy

Field emission scanning electron microscopy (FE-SEM) observations were performed on a Hitachi S-4700 microscope operated at an accelerating voltage of 20.0 kV. The samples were coated in Au with a EMscope SC500 with and intensity of 20 mA and a coating time of 1 minute.

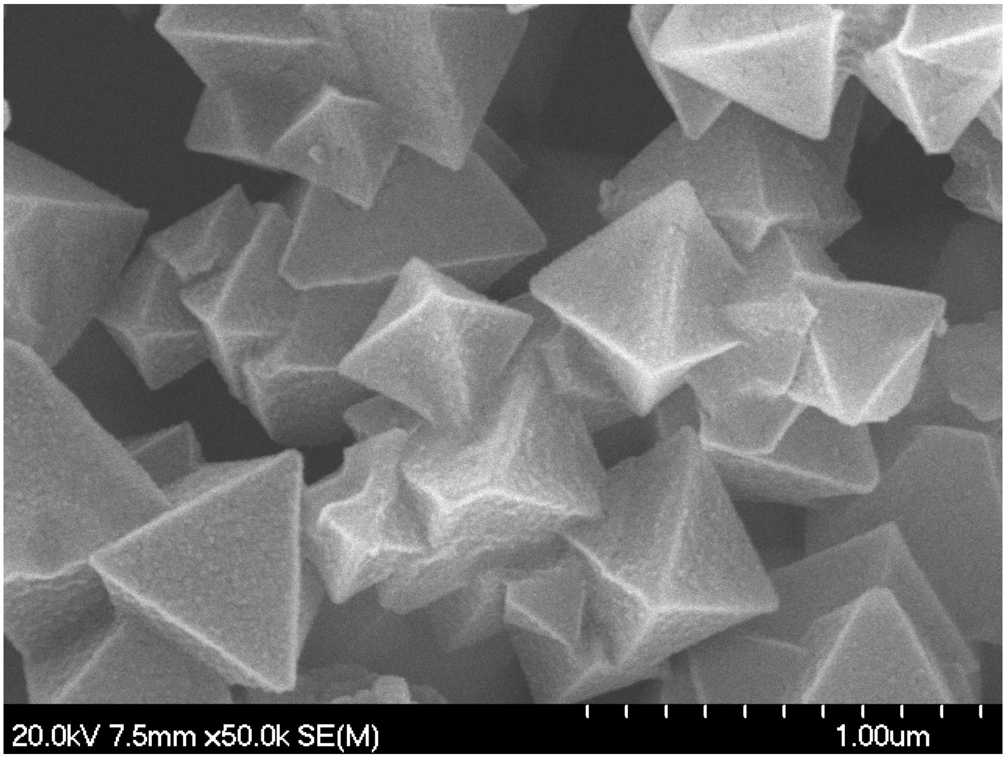

Figure S32: FE-SEM image of **MOF-808-P**.

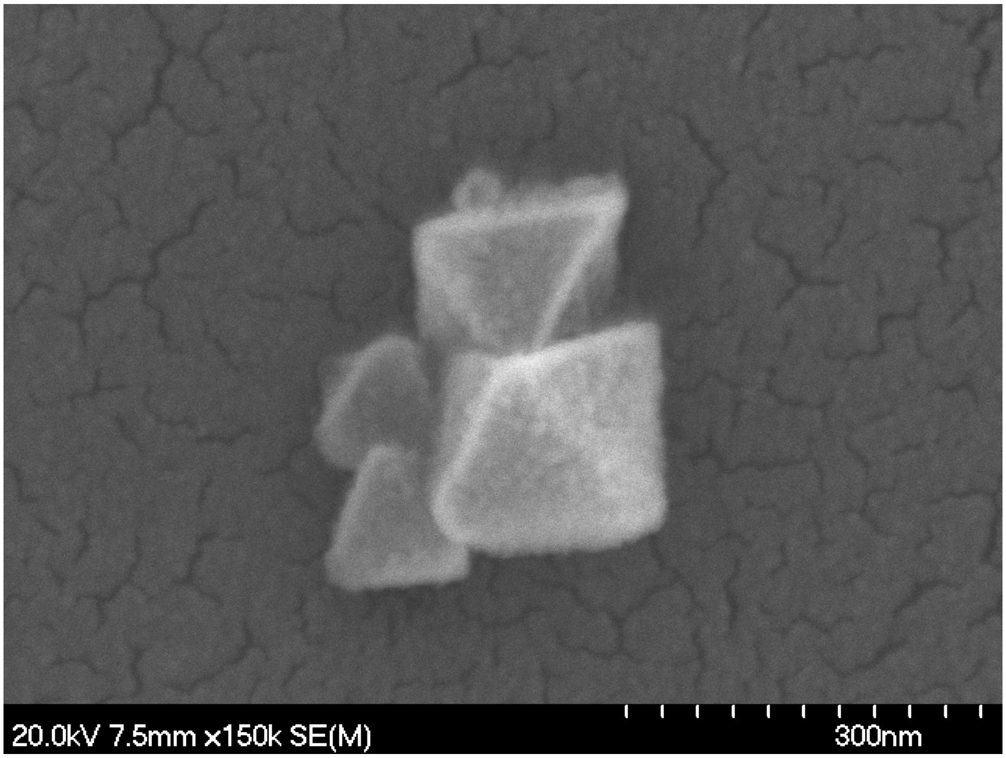

Figure S33: FE-SEM image of **2,3-DHBA-MOF-808**.

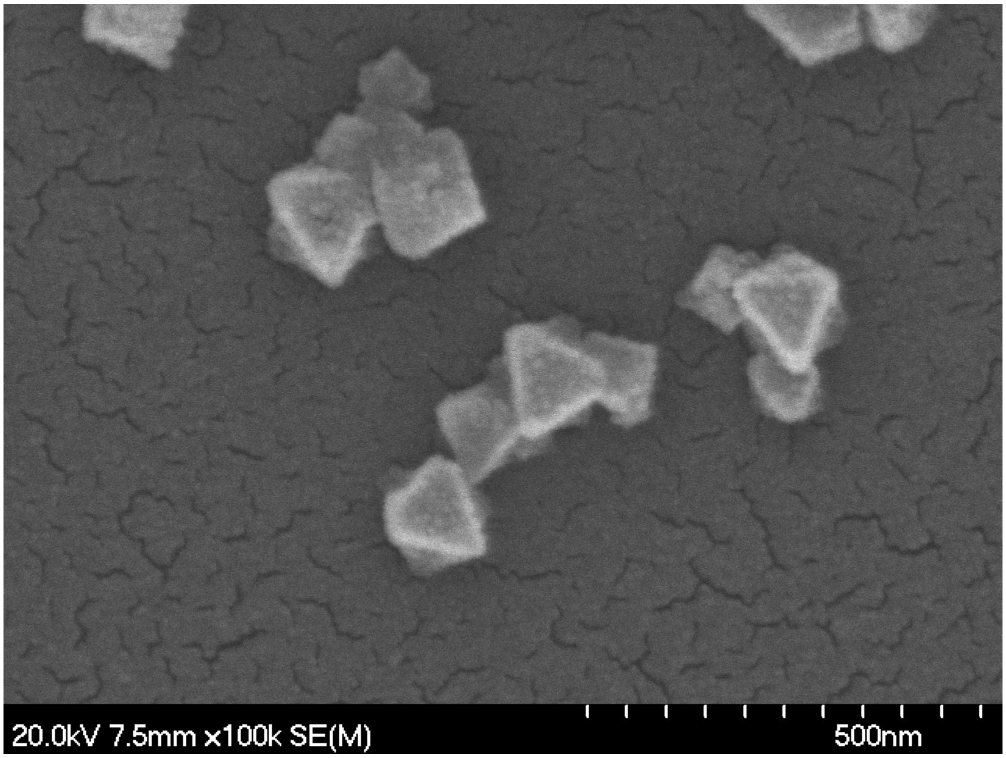

Figure S34: FE-SEM image of **3,4-DHBA-MOF-808**.

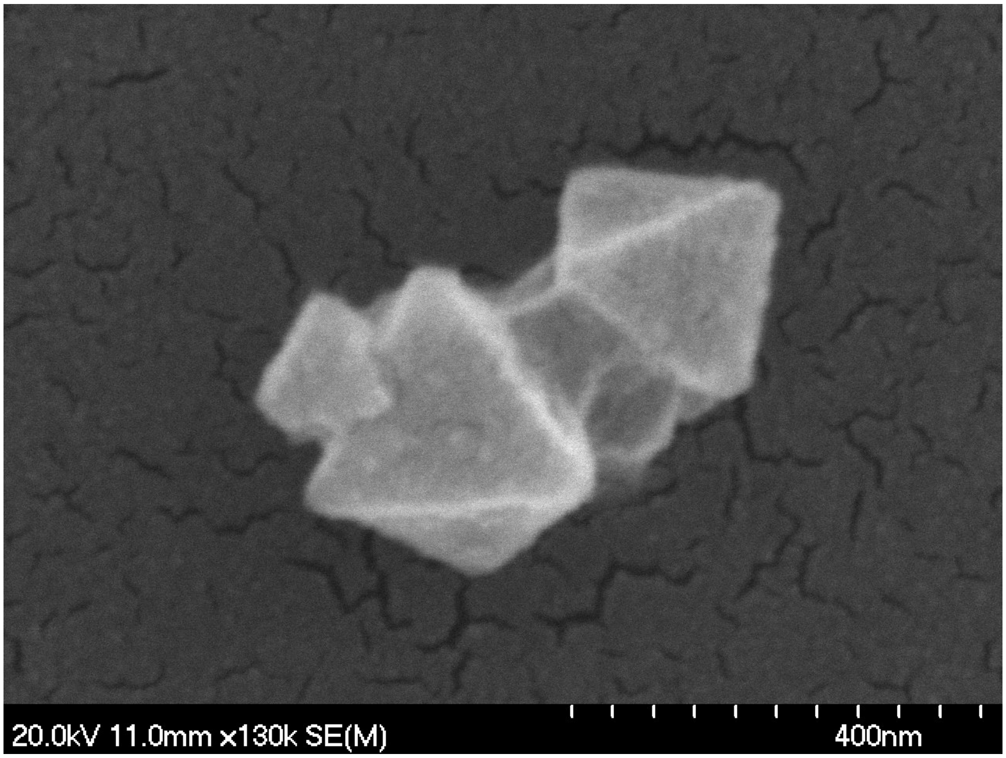

Figure S35: FE-SEM image of **Cu-2,3-DHBA-MOF-808**.

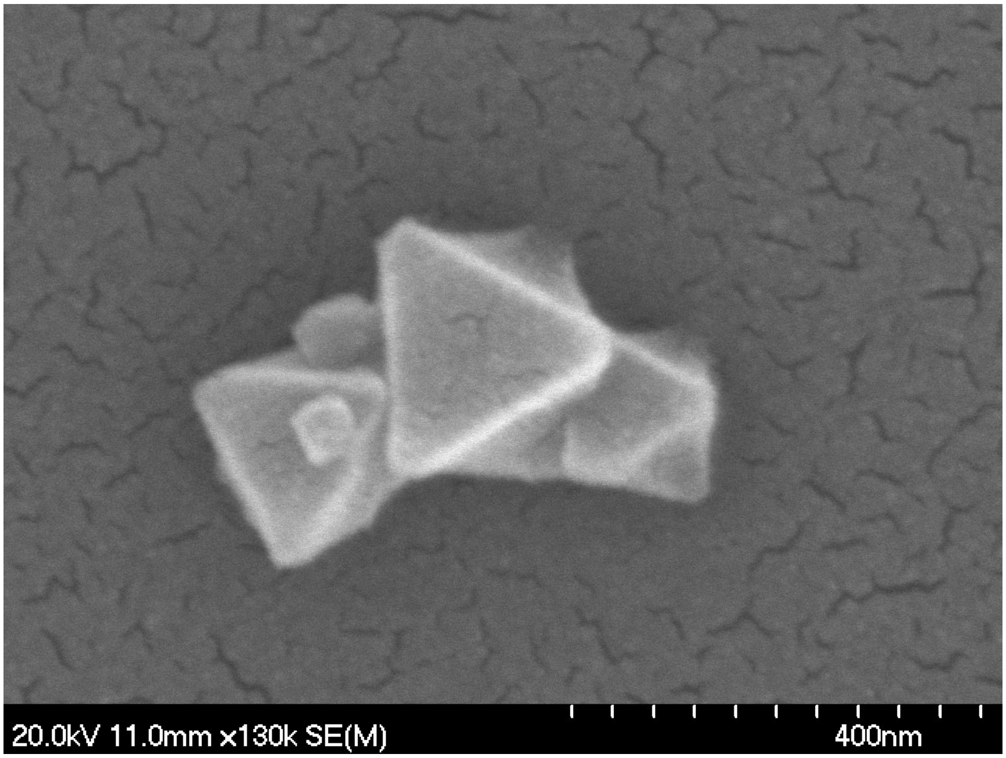

Figure S36: FE-SEM image of **Cu-3,4-DHBA-MOF-808**.

S7 Metalation studies

Inductively coupled plasma - optical emission spectroscopy (ICP-OES) analyses were performed with a Plasma Emission Spectrometer ICP Perkin Elmer model OPTIMA 2100 DV, using a CNHS Perkin Elmer 2400 Elemental Analyzer. 3 mg of sample was digested in 0.5 ml of a 1:1 H_2SO_4:H_2O_2 mixture (v:v) and taken to a 10 mL volume with distilled water in a volumetric flask.

Table S3: ICP analyses of metallation of DHBA-MOF-808.

**Metal**	**Material**	**Concentration** / ppm	**M/Zr ratio**	**M per cluster**	**Loading efficiency**
Cu	3,4-DHBA-MOF-808	Zr 63.37<br>Cu 19.77	0.445	2.67	0.89
Cu	2,3-DHBA-MOF-808	Zr 72.41<br>Cu 21.22	0.402	2.52	0.84
Fe	3,4-DHBA-MOF-808	Zr 46.58<br>Fe 10.08	0.355	2.13	1.06
Fe	2,3-DHBA-MOF-808	Zr 44.36<br>Fe 7.9	0.292	1.75	0.87
Mn	3,4-DHBA-MOF-808	Zr 99.44<br>Mn 19.86	0.331	2.00	0.66
Hg	3,4-DHBA-MOF-808	Zr 82.09<br>Hg 39.03	0.216	1.29	0.43
Ni	3,4-DHBA-MOF-808	Zr 54.10<br>Ni 15.90	0.456	2.74	0.91
Co	3,4-DHBA-MOF-808	Zr 100.1<br>Co 22.9	0.745	4.47	1.49

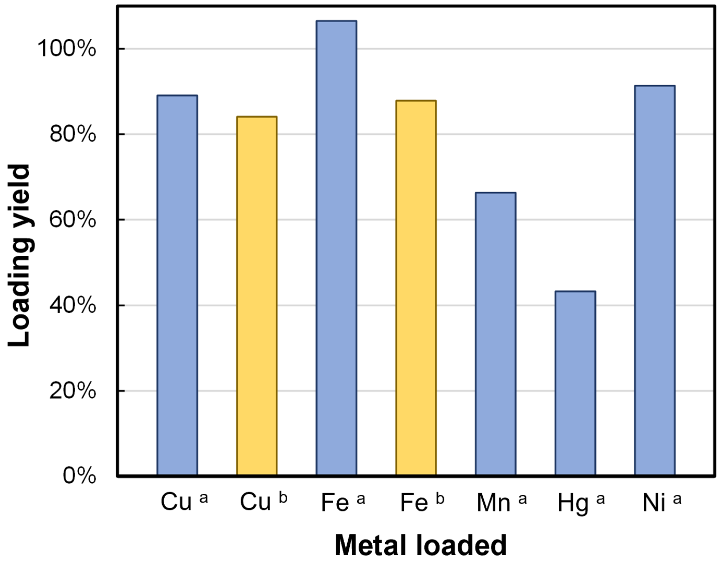

Figure S37: Efficiency of metalation for (b) **Cu-2,3-DHBA-MOF-808** (yellow) and (a) **Cu-3,4-DHBA-MOF-808**(blue).

S8 X-ray pair distribution function analyses

X-ray pair distribution function (PDF) analyses. Synchrotron X-ray total scattering data suitable for PDF analyses were collected at the P02.1 beamline[S3] at PETRA IIII (Deutsches Elektronen-Synchrotron) at beamline P02.1 on beamtimes I-20190208-EC and I-20190239-EC. Samples were ground into a fine powder and loaded into kapton capillaries (0.7 mm i.d.) and sealed. Data were collected using a Perkin Elmer XRD1621 CN3 - EHS. Empty capillary and background total scattering data were corrected for in data processing. Sample data were processed to a Qmax of 20 $Å^{-1}$ (λ = 0.2066 Å, 60 keV). Additional data was collected collected at Diamond Light Source UK, beamline I15-1, on beamtime CY28223. Samples were ground into borosilicate capillaries (1 mm i.d.) and flame-sealed. Data scans were collected during 10 minutes. Empty capillary and background total scattering data were corrected for in data processing. Sample data were processed to a Qmax of 22 $Å^{-1}$ (λ = 0.1616 Å, 76 keV). Geometric corrections and reduction to 1D data used DAWN Science software.[S4]

Differential pair distribution function (dPDF) data were obtained by subtraction of a control PDF data of MOF-808 to that of the functionalized MOF-808 sample. These analyses were performed in real-space, after applying a normalisation factor to the total PDF data.

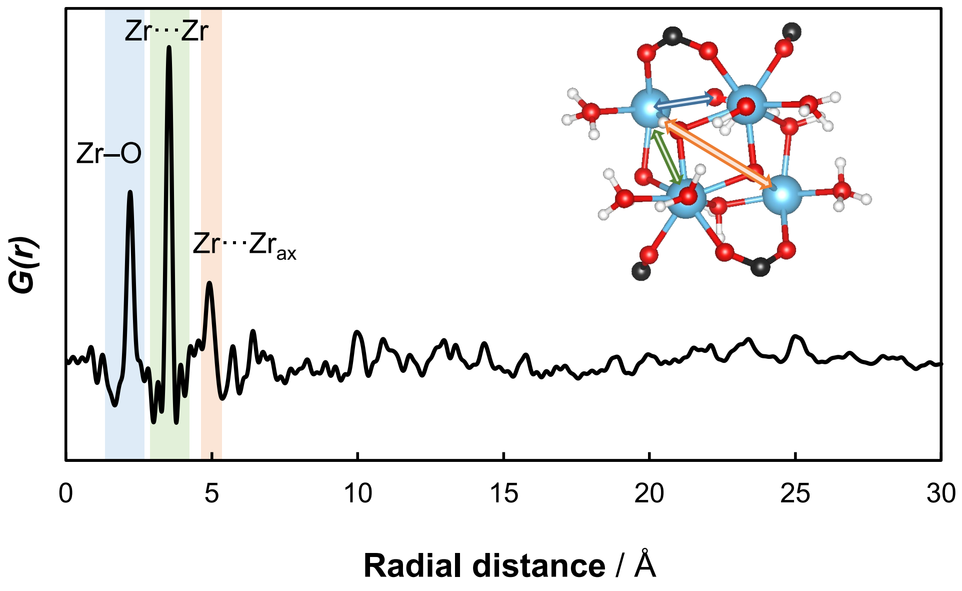

Figure S38: Total PDF data of **MOF-808-F** measured at P02.1 beamline, PETRAIII.

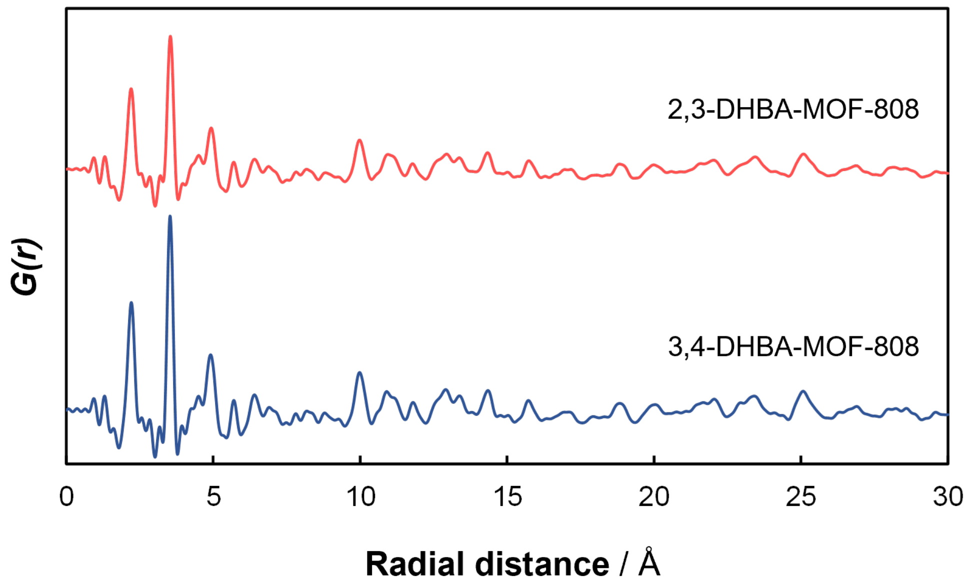

Figure S39: Total PDF data of **3,4-DHBA-MOF-808** (blue) and **2,3-DHBA-MOF-808** (red) measured at P02.1 beamline, PETRAIII.

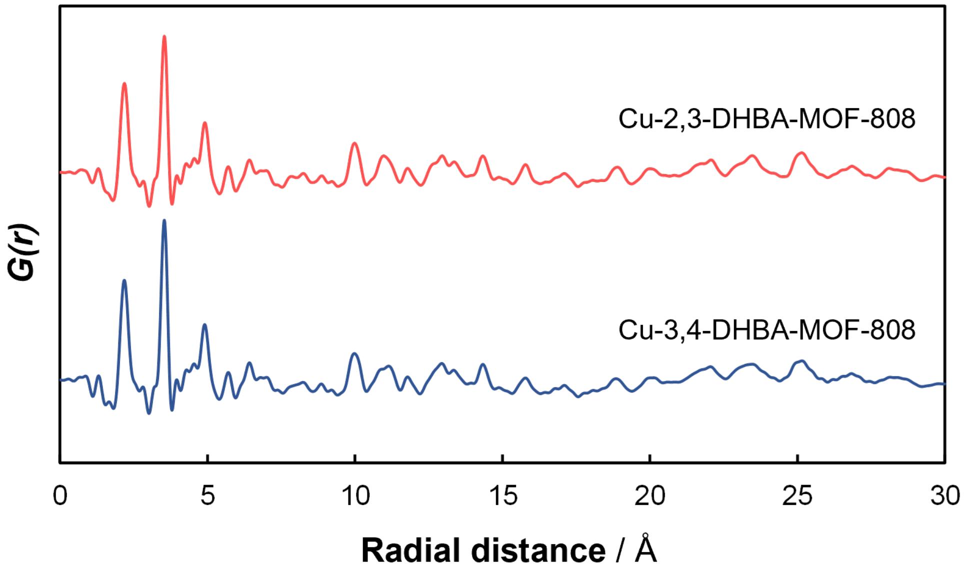

Figure S40: Total PDF data of **Cu-3,4-DHBA-MOF-808** (blue) and **Cu-2,3-DHBA-MOF-808** (red) measured at P02.1 beamline, PETRAIII.

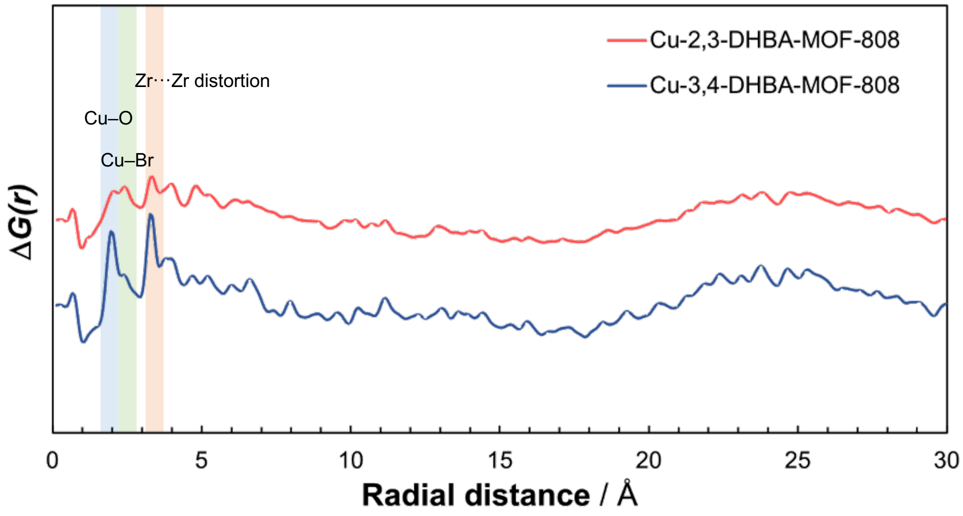

Figure S41: Differential PDF data of **Cu-3,4-DHBA-MOF-808** (blue) and **Cu-2,3-DHBA-MOF-808** (red) measured at P02.1 beamline, PETRAIII.

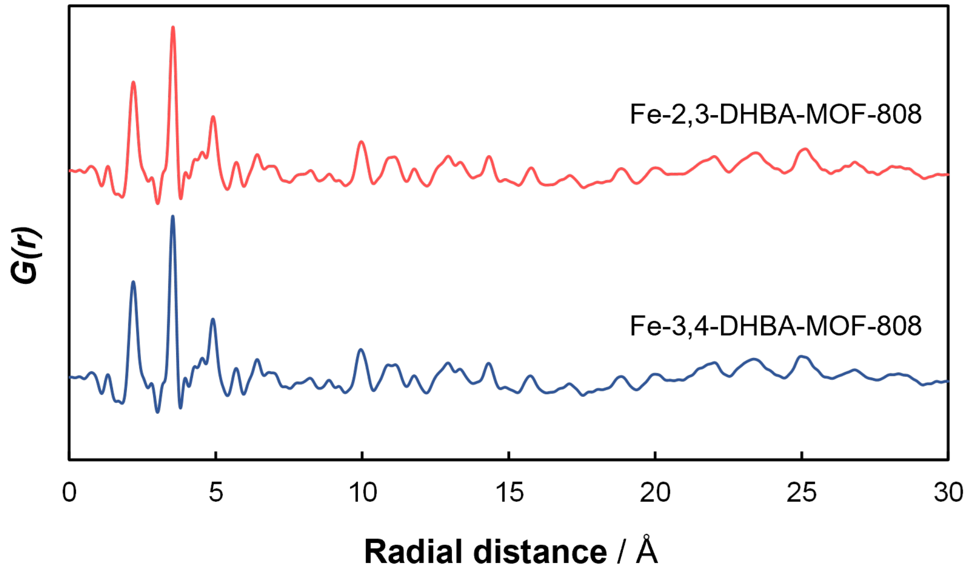

Figure S42: Total PDF data of **Fe-3,4-DHBA-MOF-808** (blue) and **Fe-2,3-DHBA-MOF-808** (red) measured at I15-1 beamline, Diamond Light Source.

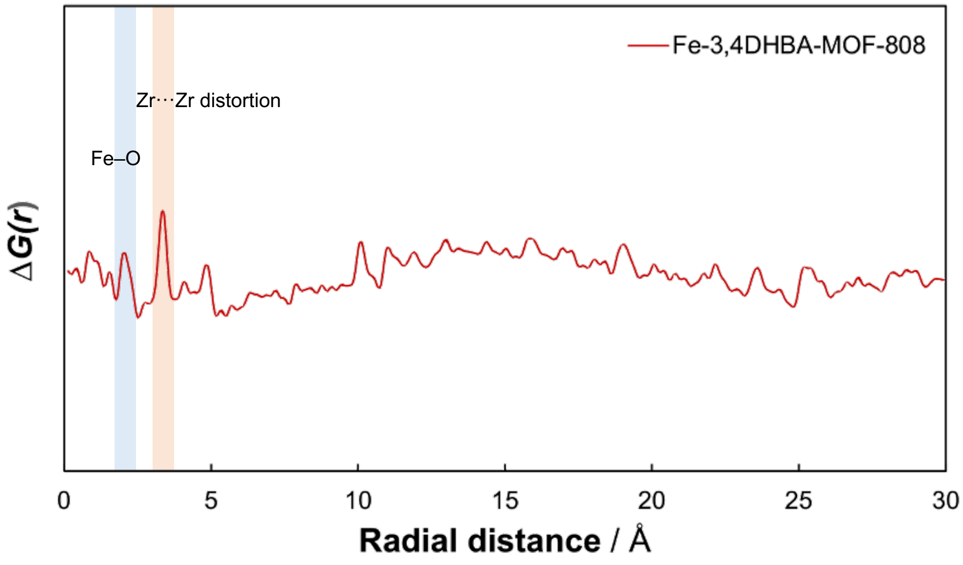

Figure S43: Differential PDF data of **Fe-3,4-DHBA-MOF-808** (blue) and **Fe-2,3-DHBA-MOF-808** (red) measured at the I15-1 beamline, Diamond Light Source.

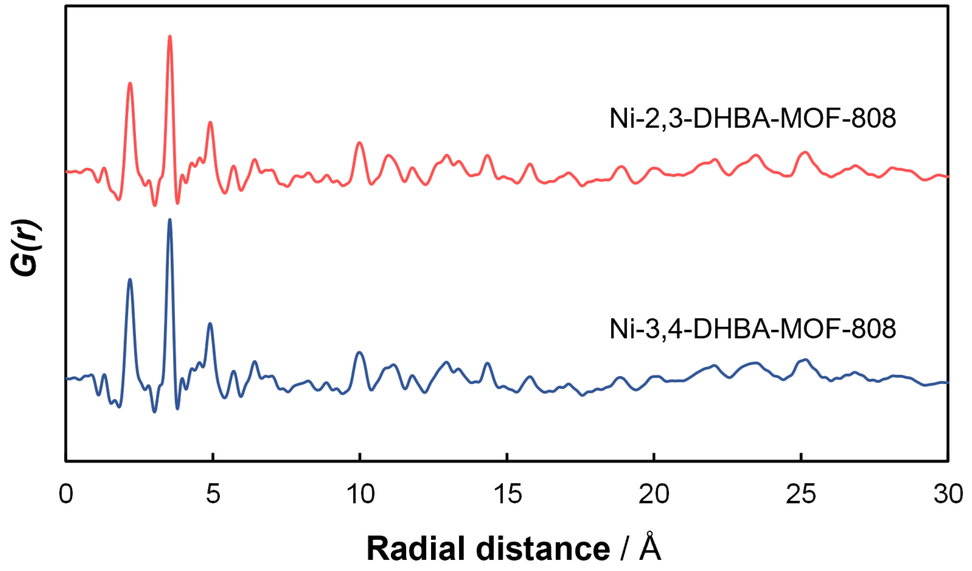

Figure S44: Total PDF data of **Ni-3,4-DHBA-MOF-808** (blue) and **Ni-2,3-DHBA-MOF-808** (red)) measured at P02.1 beamline, PETRAIII.

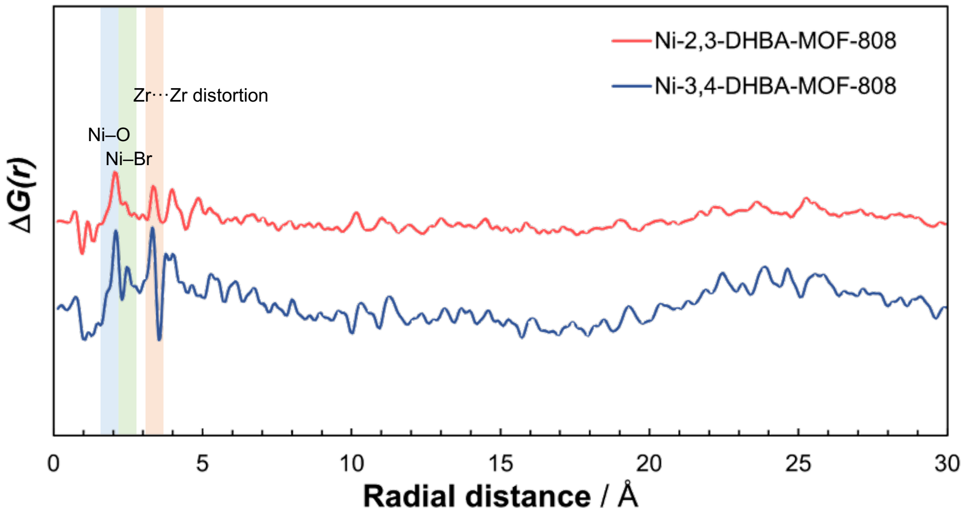

Figure S45: Differential PDF data of **Ni-3,4-DHBA-MOF-808** (blue) and **Ni-2,3-DHBA-MOF-808** (red) measured at P02.1 beamline, PETRAIII.

S9 Nuclear magnetic resonance spectra

^{1}H Nuclear Magnetic Resonance (^{1}H-NMR) spectra were recorded in DMSO-d_6 or $CDCl_3$ solution on a 300 MHz Bruker Advance II NMR spectrometer. Chemical shifts are reported in parts per million (ppm), referenced to the solvent peak, DMSO-d_6 defined at δ = 2.50 ppm, or $CDCl_3$ defined at δ = 7.26 ppm. ^{1}H-NMR splitting patterns were designated as: singlet (s), doublet (d), triplet (t), quadruple (q), quintet (p) and double doublet (dd). Signals that could not be easily interpreted or visualized were designated as multiplet (m). Coupling constants (J) are indicated in Hz.

General procedures for MOF-808 digestion.

1. 10 mg of the MOF is suspended in DMSO-d_6 (700 μL). A drop (20 μL) of HF 50% in water was added to the solution, and a drop (20 μL) of deuterated water (D_2O). The reaction is undisturbed for 16 h.

2. 10 mg of the MOF is suspended in DMSO-d_6 (700 μL). A drop (20 μL) of D_2SO_4 was added to the solution. The reaction is undisturbed for 16 h.

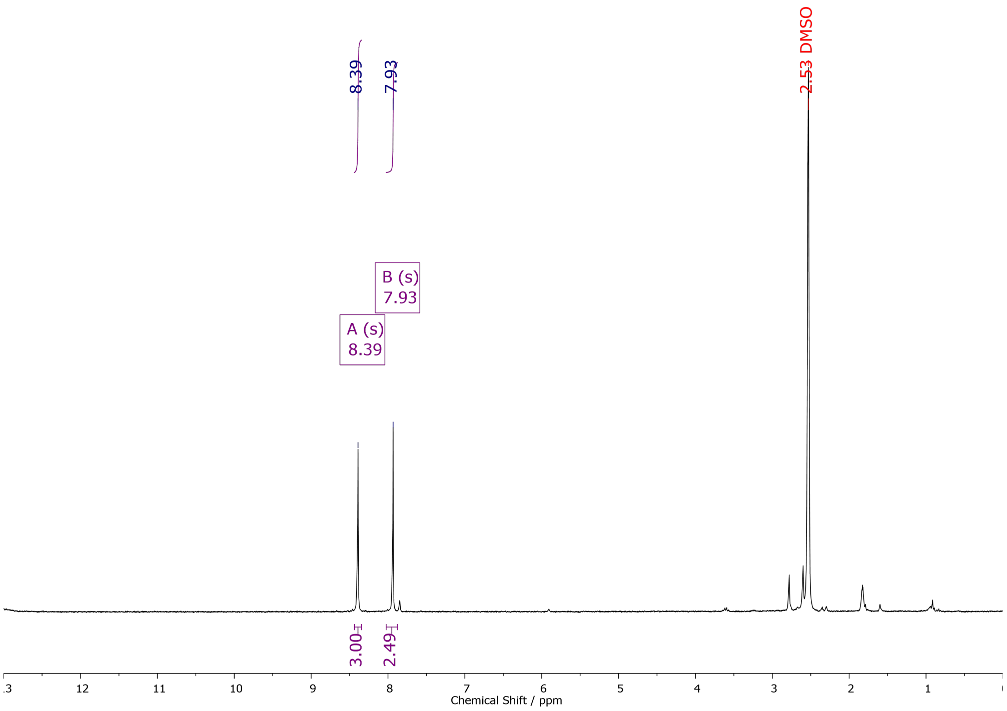

Figure S46: ^{1}H-NMR of **MOF-808-F** in DMSO-d_6:D_2SO_4.

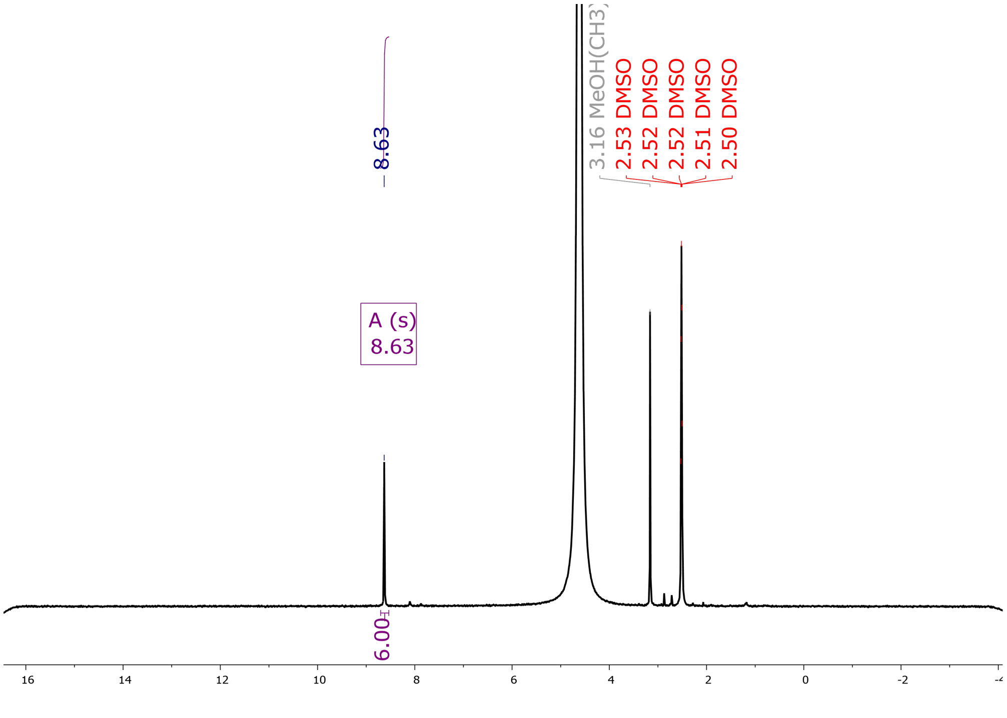

Figure S47: ^{1}H-NMR of **MOF-808-P** in DMSO-d_6-HF:D_2O.

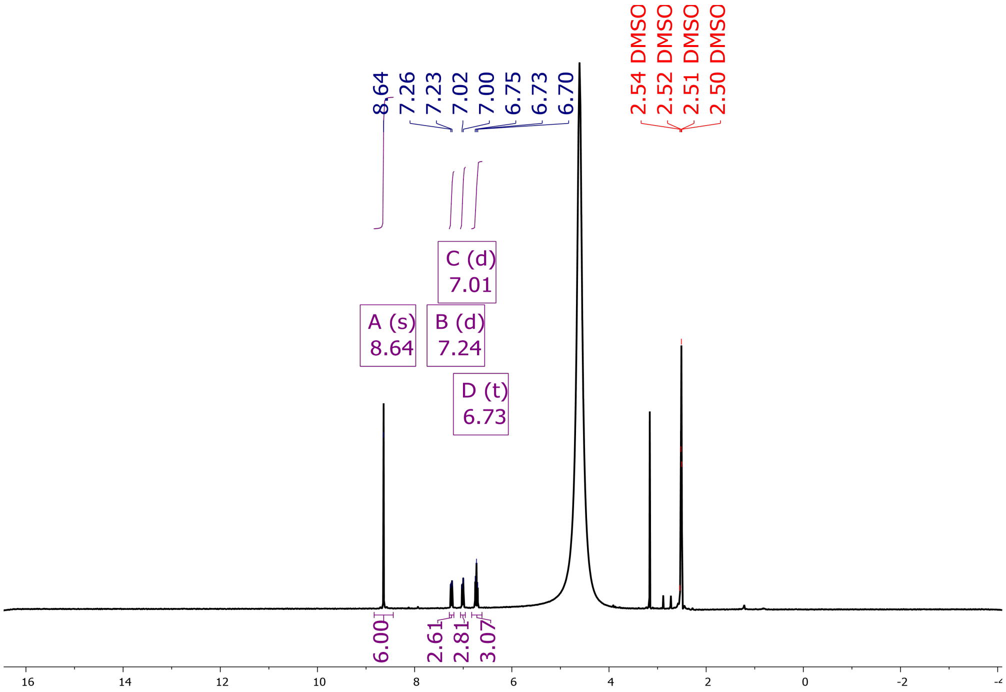

Figure S48: ¹H-NMR of **2,3-DHBA-MOF-808** in DMSO-d_6-HF:D_2O.

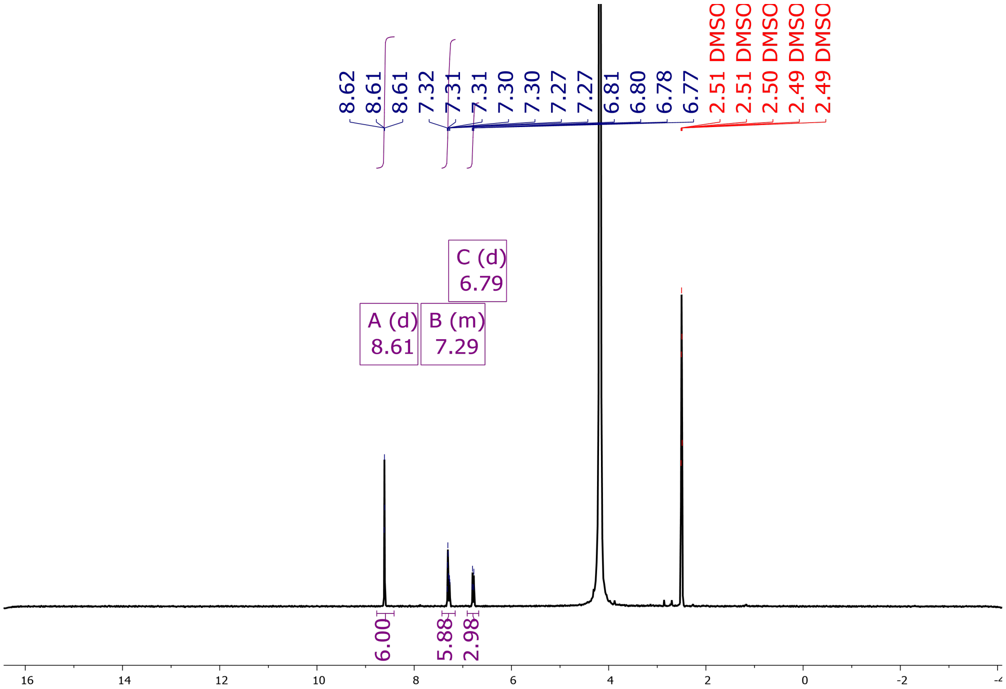

Figure S49: ¹H-NMR of **3,4-DHBA-MOF-808** in DMSO-d_6-HF:D_2O.

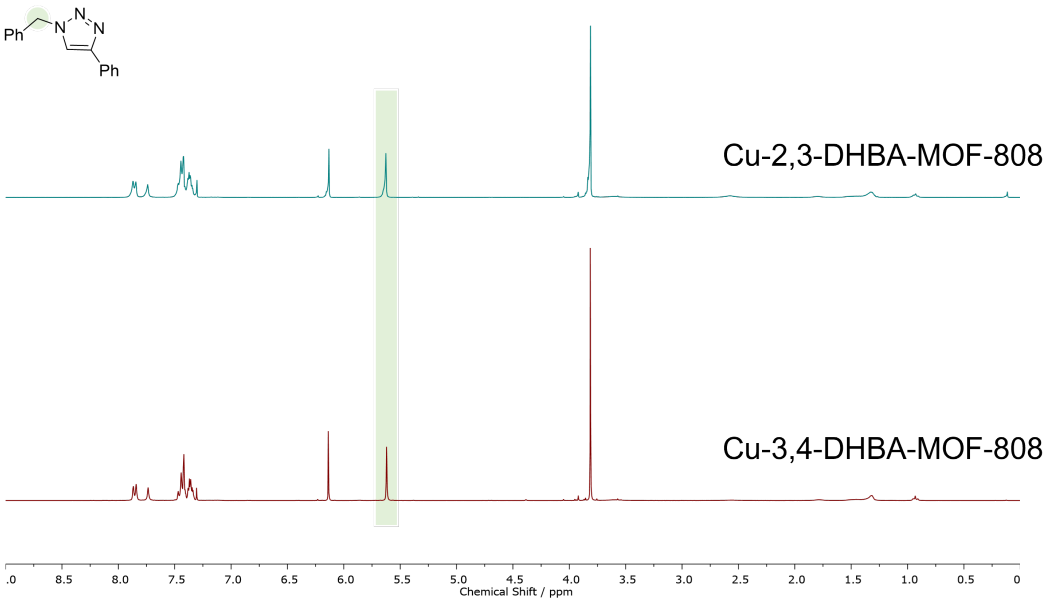

Figure S50: ^{1}H-NMR of 1-benzyl-4-phenyl-1,2,3-triazole from **Cu-2,3-DHBA-MOF-808** (blue) and **Cu-3,4-DHBA-MOF-808** (red) catalytic reactions in $CDCl_3$.

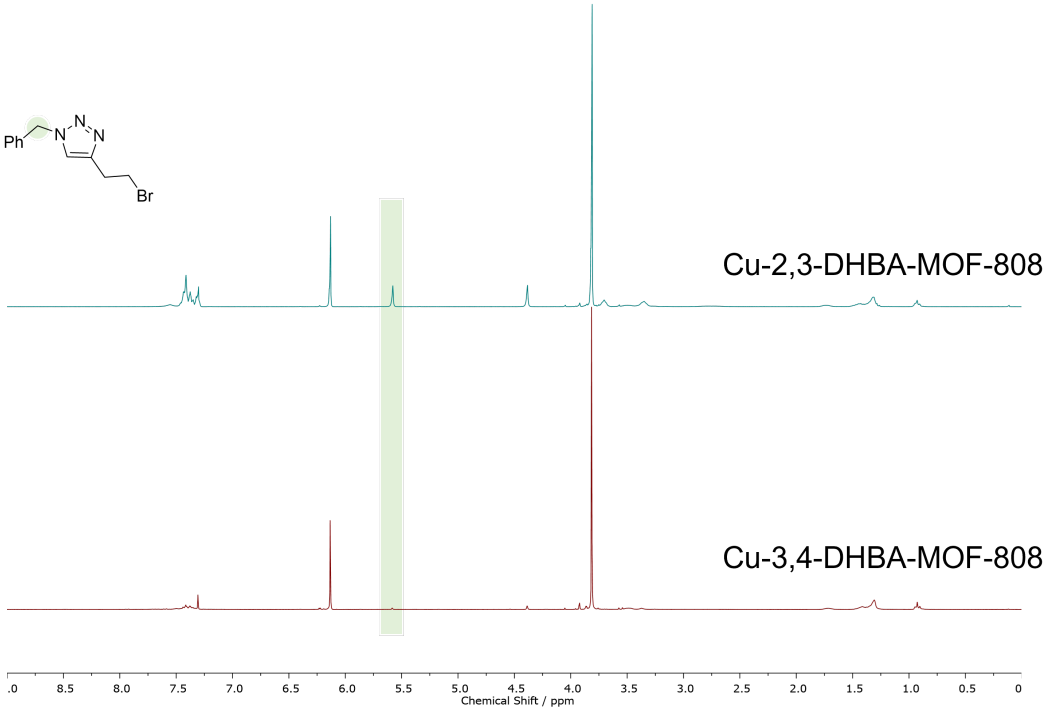

Figure S51: ^{1}H-NMR of 1-benzyl-4-(2-bromoethyl)-1,2,3-triazole from **Cu-2,3-DHBA-MOF-808** (blue) and **Cu-3,4-DHBA-MOF-808** (red) catalytic reactions in DMSO-d_6-HF:D_2O.

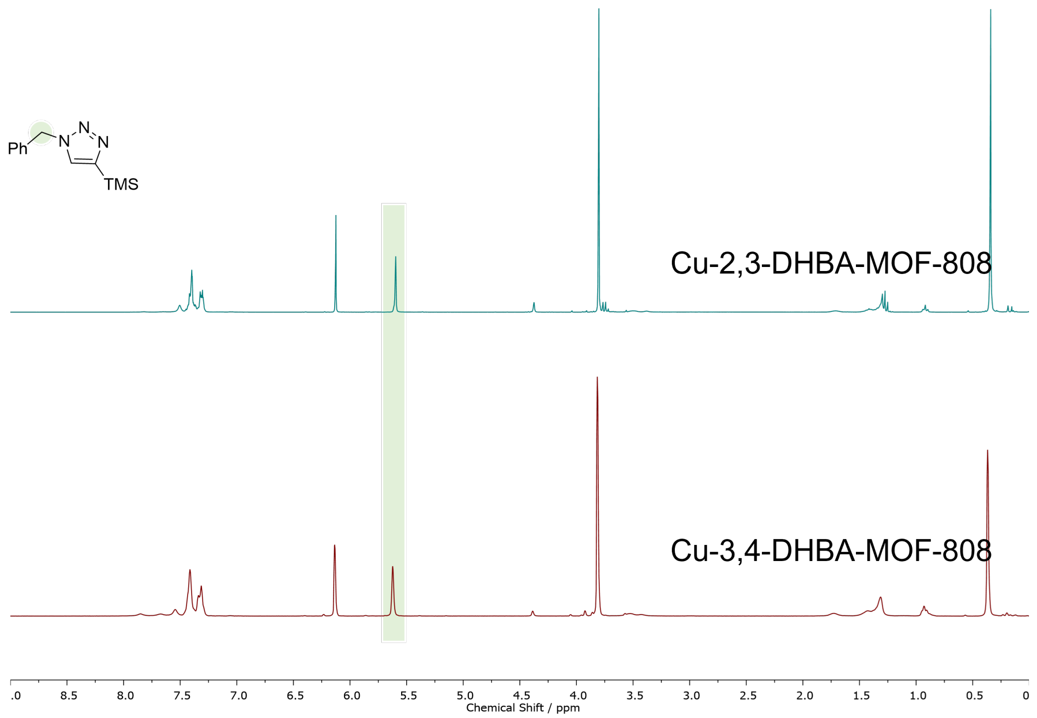

Figure S52: ^{1}H-NMR of 1-benzyl-4-trimethylsilyl-1,2,3-triazole from **Cu-2,3-DHBA-MOF-808** (blue) and **Cu-3,4-DHBA-MOF-808** (red) catalytic reactions in $CDCl_3$.

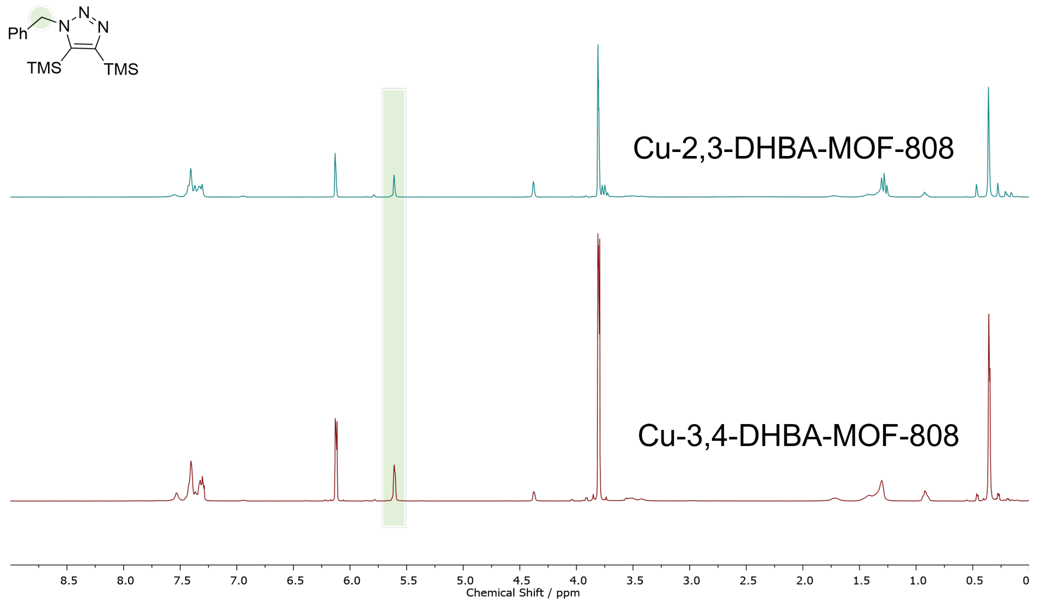

Figure S53: ^{1}H-NMR of 1-benzyl-4,5-bis(trimethylsilyl)-1,2,3-triazole from **Cu-2,3-DHBA-MOF-808** (blue) and **Cu-3,4-DHBA-MOF-808** (red) catalytic reactions in $CDCl_3$.

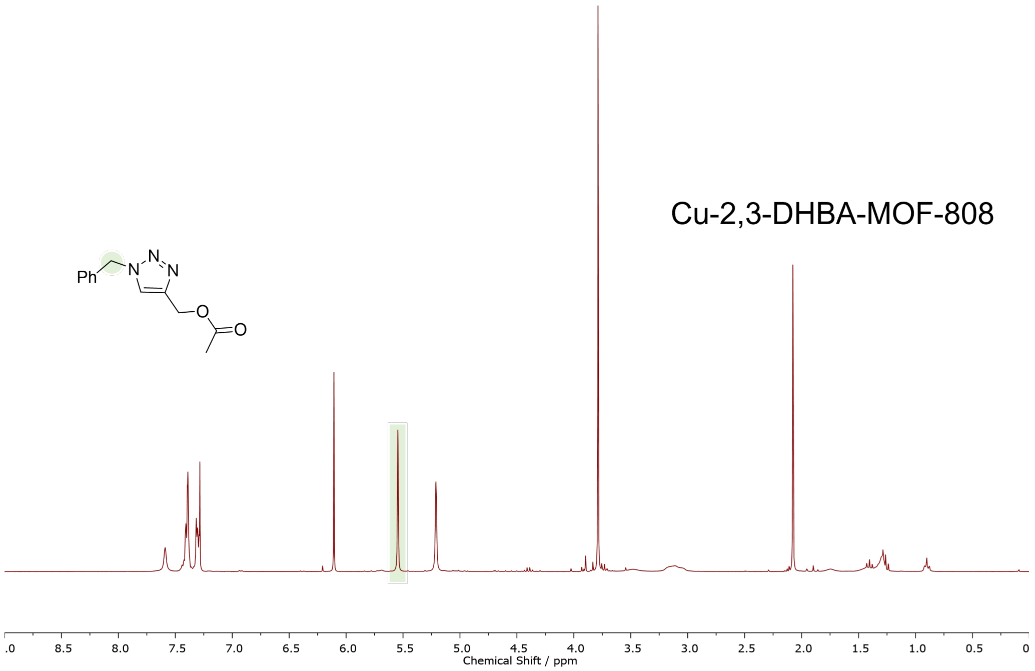

Figure S54: ^{1}H-NMR of (1-benzyl-1H-1,2,3-triazol-4-yl)methyl acetate from **Cu-2,3-DHBA-MOF-808** catalytic reaction in $CDCl_3$.

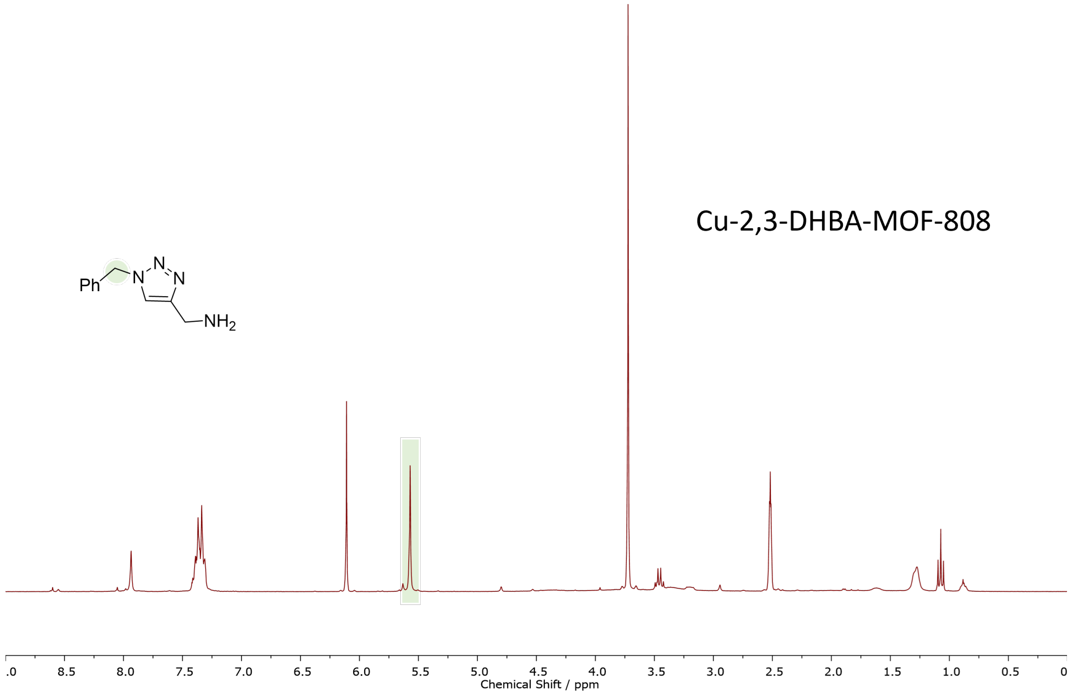

Figure S55: ^{1}H-NMR of 1-benzyl-4-(aminomethyl)-1,2,3-triazole from **Cu-2,3-DHBA-MOF-808** catalytic reaction in DMSO-d_6.

S10 Catalytic tests

1,3-dipolar cycloaddition. 5 mg of Cu-DHBA-MOF-808 were added to a stirred solution of benzyl azide (100 μL, 0.8 mmol, 1 eq) and the corresponding alkyne (0.91 mmol, 1.15 eq) in dry and deoxigenated EtOH (5 mL). The reaction mixture was heated at 70 °C for 16 h. Then the reaction was filtered thought a 0.22 μm nylon syringe filter, and 1,3,5-trimethoxybenze (61 mg, 0.4 mol, 0.5 eq) was added to the reaction mixture. The solvent is removed *in vacuo*. The resulting solid is dissolved in ethyl acetate, and washed with water and brine. The organic layers are separated, dried and the solvent is removed *in vacuo*. The reaction yield is quantified by quantitative (qNMR) using 1,3,5-trimethoxybenze as internal standard.

Recyclability tests 5 mg of Cu-DHBA-MOF-808 were added to a stirred solution of benzyl(100 μL, 0.8 mmol, 1 eq) and phenylacetylene (0.91 mmol, 1.15 eq) in dry and deoxigenated EtOH. The reaction mixture was heated at 70 °C for 16 h. Then the reaction was filtered centrifuged and the MOF separated. 1,3,5-trimethoxybenze (61 mg, 0.4 mol, 0.5 eq) was added to the reaction mixture. The reaction yield is quantified by quantitative (qNMR) using 1,3,5-trimethoxybenze as internal standard. The MOF is washed with water and methylenchloride to remove any trace of salt, reagent, or product. The product is oven-dried at 50°C overnight yields the clean catalytic material.

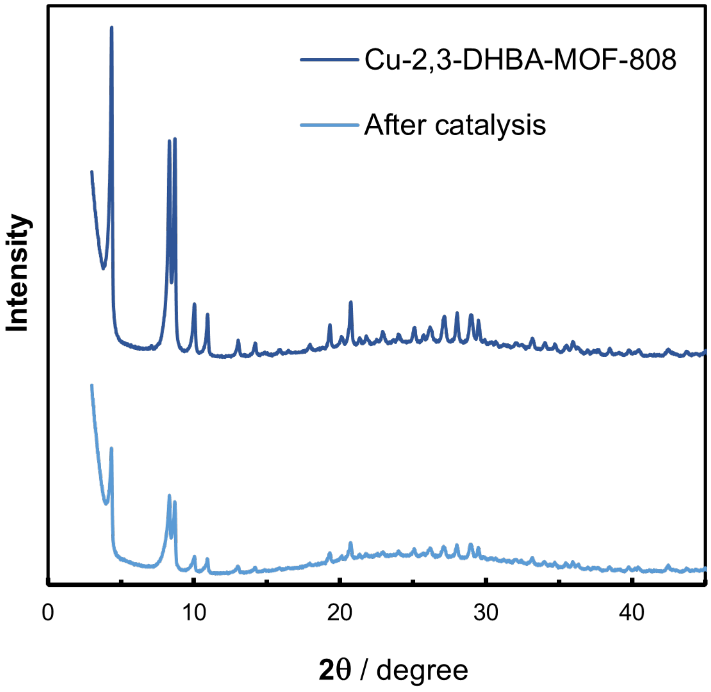

Figure S56: PXRD data of Cu-2,3-DHBA-MOF-808 before and after catalysis.

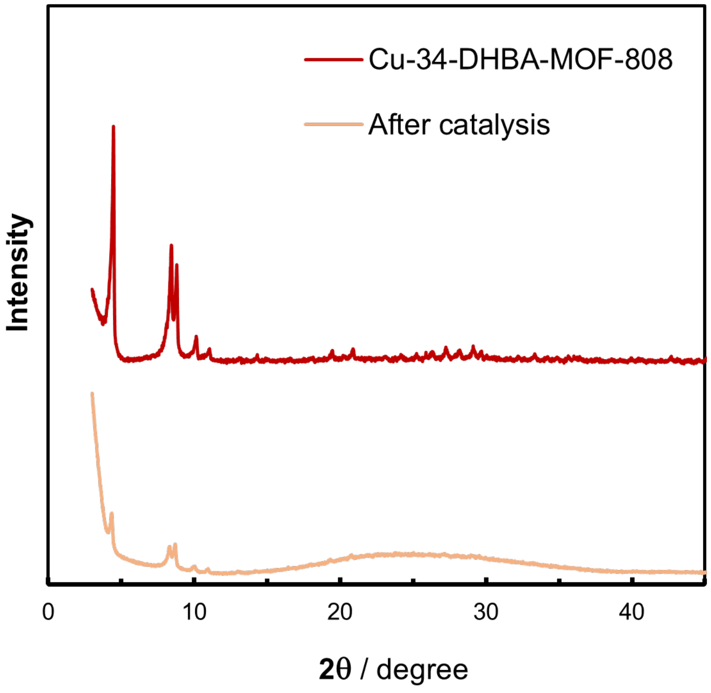

Figure S57: PXRD data of Cu-3,4-DHBA-MOF-808 before and after catalysis.

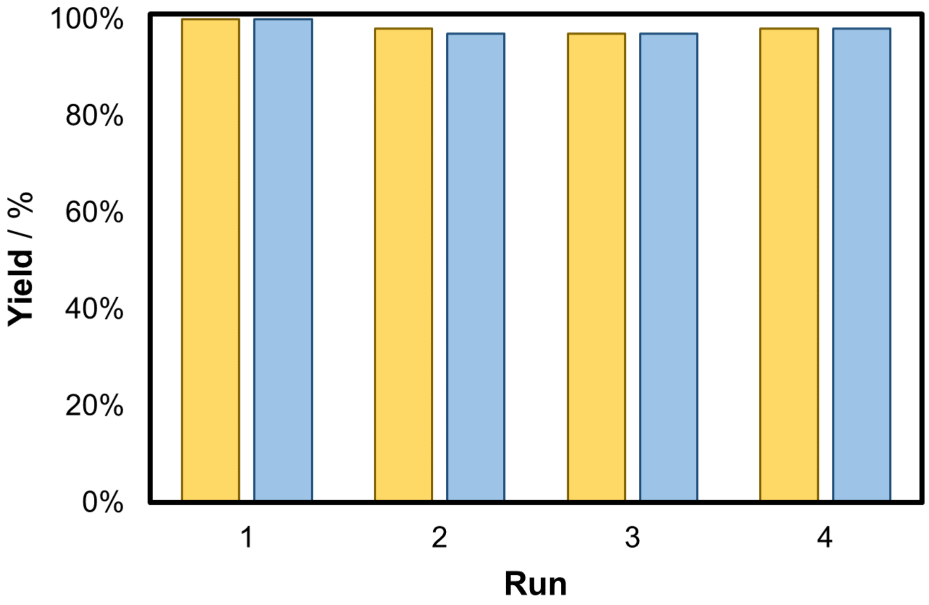

Figure S58: Yield of product **1** by using (b) **Cu-2,3-DHBA-MOF-808** (yellow) and (a) **Cu-3,4-DHBA-MOF-808**(blue) as catalyst in different runs

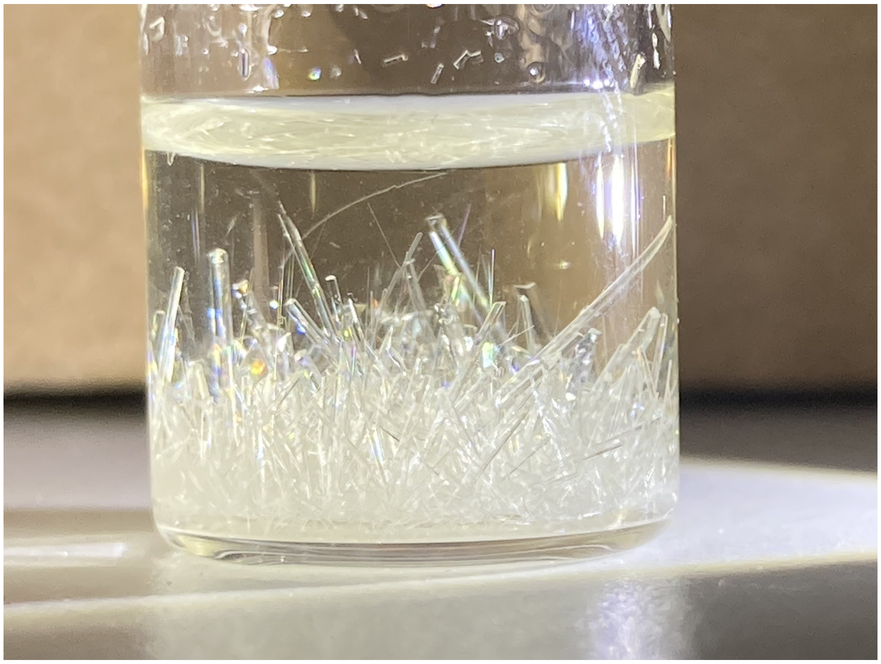

Figure S59: Crystals of 1-benzyl-4-phenyl-1,2,3-triazole.

S11 Computational details

Our analysis of the structural and vibrational properties of MOF-808 is carried out using plane waves calculations based on Density Functional Theory (DFT). In particular, we have employed the VASP code[S5] with an energy cutoff of 425 eV for the plane-wave basis set, pseudopotentials built with the projector augmented wave method[S6,S7] and the Perdew-Burke-Ernzerhof exchange and correlation functional[S8] in combination with the D3 semi-empirical correction.[S9] This last correction is important to account for the non-local van der Waals interactions, which usually play a non-negligible role in most of the adsorption processes.

The cubic crystal structure of MOF-808 (space group no. 227, Fd$\overline{3}$m) can be viewed as an arrangement of $[Zr_6O_8H_4]^{12+}$ clusters linked by BTC ligands.[S10] These molecules are bonded to the metallic clusters through their terminal carboxylate groups in such a way that all carboxylate groups act as bidentate ligands. Unfortunately, the convectional unit cell contains over one thousand atoms, which prevents the use of quantum first-principles calculations with standard 3D periodic boundary conditions. Even considering a primitive cell, it contains a few hundreds of atoms, still too large to perform a normal mode analysis and the subsequent production of vibrational spectra at quantum level, even in the pristine MOF. To overcome this issue, we have used a cluster approach using one single $[Zr_6O_8H_4]^{12+}$ unit extracted from the crystal structure and coordinated by six BTC ligands. The distal carboxylate groups were protonated to avoid a spurious electron charge accumulation. The whole structure was placed on a cubic unit cell with a 33 Å edge in such a way that the distance between periodical imagines is minimised and leaving room enough to introduce the ligands of the functionalisation. This fragment, that we will discuss in depth in the next section, allows us to study the main structural and vibrational properties pristine MOF-808 and the subsequent functionalisation with 2,3- and 3,4-DHBA acid ligands.

All equilibrium geometries were obtained in our cluster models using a conjugate gradient algorithm in combination with electronic self-consistent loops. The self-consistent electronic

loops were converged with a tolerance better than 10^{-6} eV and the stopping criterion for the structural optimization was that forces upon atoms had to be smaller than 0.005 eV/Å. This strict criterion in forces is needed to ensure the success of the subsequent normal mode analysis, where a true energy minimum is required. Otherwise, spurious imaginary modes may appear. During the structural relaxations the oxygen and hydrogen atoms belonging to the distal carboxylate groups were fixed on their bulk positions to reproduce the real MOF environment while all the rest atoms were free to relax in order to find their equilibrium positions.

The vibrational properties of pristine and functionalized MOF-808 were investigated following a normal mode analysis under the classical harmonic approximation. This approach consists of the direct diagonalization of the mass-weighted Hessian matrix that appears when solving the atomic equations of motion using normal coordinates.[S11,S12] The Hessian matrix is numerically obtained by means of finite differences considering a total of six displacements of ±0.02 Å per atom along the three Cartesian coordinates.

Once the normal modes (eigenvectors and eigenfrequencies) are known, the IR intensity of each mode is estimated as described in detail in previous work.[S13] Essentially, the IR intensity of the nth normal mode, I_n^{IR}, is related to the square of the variation of the electric dipole moment along the vibration.[S11] In turn, it depends on the so-called Born effective charges $Z^*_{\alpha\beta,\tau}$ as[S14]

$$I_n^{\mathrm{IR}} \propto \sum_{\alpha=1}^{3} \left| \sum_{\beta=1}^{3} \sum_{\tau=1}^{N} Z^*_{\alpha\beta,\tau} A^{\tau}_{\beta,n} \right|^2 , \tag{S1}$$

where N is the number of atoms in our system and $A^{\tau}_{\beta,n}$ is the nth eigenvector. Notice that the Born effective charges can be directly calculated within DFT methods in the frame of the linear response theory using Density Functional Perturbation Theory (DFPT).[S15,S16] After collecting the IR intensities of all individual modes, a complete IR spectrum for the system can be generated as a sum of continuous Lorentzian functions centered in each eigenfrequency. A smearing of 3 cm^{-1} is considered for the Lorentzian functions because it is a balance

between a good resolution and providing an appearance similar to the experimental spectra. It is worth noting that the IR intensity obtained with Eq. (S1) is related to the absorbance (A) measurement. If we want to convert it to transmittance (T), we have to take into account that $T = 10^{-A}$, as we have made for the present work.

S12 Cluster models

We have studied the structural and vibrational properties of bare and functionalized MOF-808 following a cluster-type approach. As we pointed out in the previous section, due to the large size of the unit cell, it is impossible to calculate the vibrational modes of the whole crystal structure with periodic boundary conditions. The selected cluster model consists of a single $[Zr_6O_8H_4]^{12+}$ unit surrounded by six BTC linkers while the equatorial coordination sites are saturated with hydroxy ligands or water molecules leading to charge neutrality. Notice that the distal carboxylate groups are protonated (i.e. COOH) to avoid an unphysical charge accumulation in the simulation box. In the original crystal structure, the equatorial sites were fully saturated with formate ligands. The substitution of these formate ligads by hydroxy/water ligands leads to a slight rearrangement of the $[Zr_6O_8H_4]^{12+}$ polyhedra, inducing some variations in the Zr···Zr distances.

The experiments suggest that a number between two and four molecules of DHBA are able to bond to the equatorial coordination sites after removing the necessary hydroxy/water original ligands. Unfortunately, the X-ray based experiments cannot provide further information about the exact distribution of these new ligands due to their organic nature. In order to get more insight on this issue, we have performed several calculations on this cluster model incorporating three of these ligands. The number of ways in which these ligands can bond to the equatorial open metal sites of the $[Zr_6O_8H_4]^{12+}$ polyhedra is relatively large, but, at the same time, the high symmetry of them significantly reduce the total number. In particular, we have tried three different distributions, which are a very representative set. Although not completely equivalent, all other possibilities will be closely related to one of these distributions.

The bonding of DHBA molecules takes place through the carboxylate groups acting as bidentante ligands, similarly to the original BTC linkers contained in the pristine crystal structure. We must say that for the 2,3- isomer is spatially possible a mixed carboxylate/phenolate coordination mode similar to the one found in MOF-74. However, our calcu-

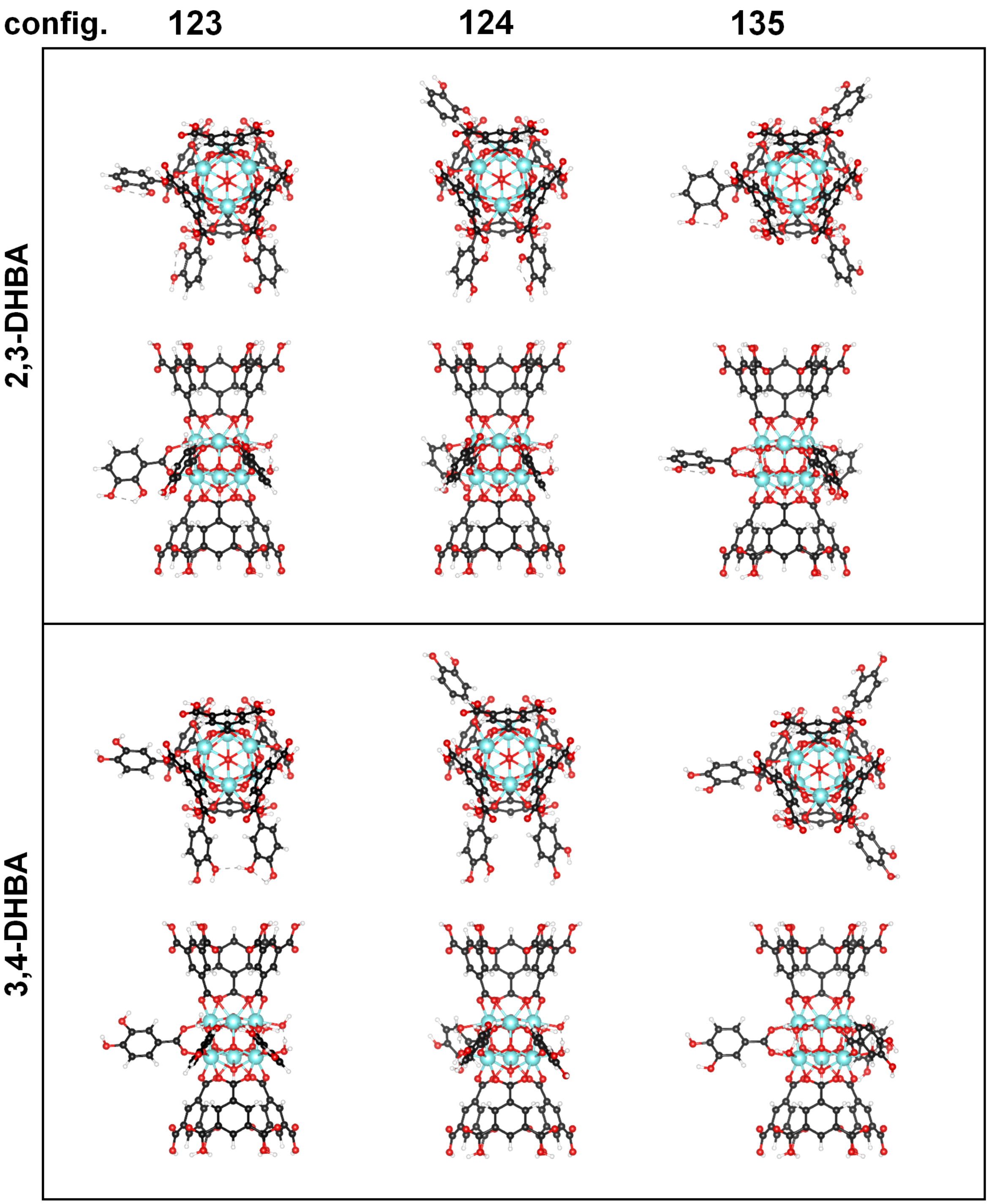

Figure S60: Ball-and-stick models of the equilibrium geometries of the MOF-808 functionalized with three molecules of 2,3- (top panel) and 3,4-DHBA (lower panel). In each case we have considered three different adsorption configurations which have a similar energetics.

lations discard this possibility because there are not stable configurations with this choice. Moreover, the stability of the $[Zr_6O_8H_4]^{12+}$ polyhedra seems to be affected proceeding this way. Thus, we restrict our analysis to the 2,3- and 3,4- functionalization through the carboxylate groups, the only one predicted as stable by our calculations. Taking into account this circumstance, there are several ways for the incorporation of the ligands, but in all of them, the carboxylate ligands must bond to two adjacent Zr metal centers. This is because the coordination to the same metal center would lead to an unnatural (too small) COO bond angle, being an unfavourable choice. Since, we have a total of twelve outer coordination sites originally occupied by hydroxy/water ligands, all possible coordination modes reduce essentially to the three possibilities depicted in figure S60. By analogy to the benzene nomenclature with three substituents, we have labelled these coordination modes as 123, 124 and 135.

Our calculations do not show a clear preference for any of these coordination modes. It is true there are slight energy differences between them and with opposite trends for 2,3- and 3,4- isomers. Namely, we find that the most stable configuration for the 2,3- isomer is 135 configuration, being the 124 and 123 slightly less energetically favourable by 0.27 eV and 0.49 eV respectively. Conversely, the 123 configuration is slightly more energetically favourable for the 3,4- isomer following the 123 $>$ 124 $>$ 135 trend. In this case the 124 and 135 configurations are 0.19 eV and 0.23 eV less stable than the most stable 123 configuration. These small energy differences arise from the eventual formation of intra or interligand H-bonds, but all the configurations display basically the same features, as it can be observed in the figure and a similar energetics.

S13 Further IR theoretical spectra

In figures S61 and S62 we show the theoretical IR spectra of functionalized MOF-808 in all the adsorption configurations that we have discussed in the previous section. All of them display basically the same IR features including the new signal at 1200 cm^{-1} arising from the new ligands. In the main text, we have included for comparison only the spectrum of the most energetically favourable configuration for each isomer.

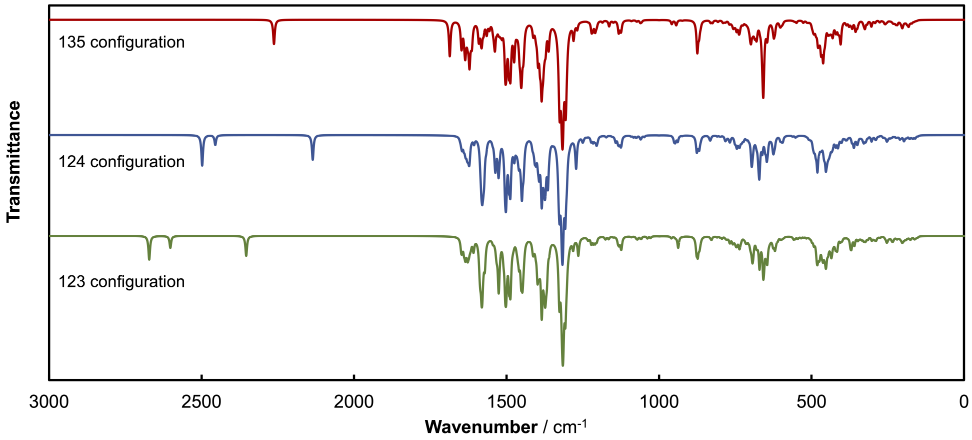

Figure S61: Theoretical IR spectra of MOF-808 functionalized with three 2,3-BHBA ligands in the equatorial plane. We show the IR spectra of the three configurations considered in this work.

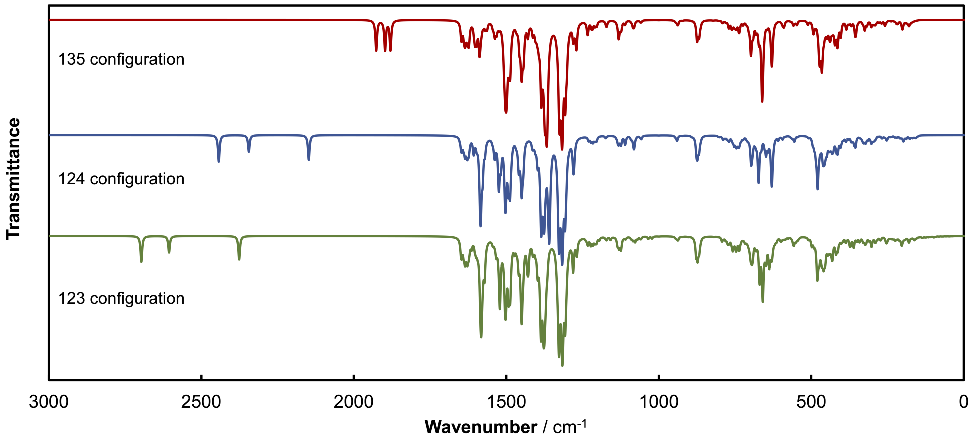

Figure S62: Theoretical IR spectra of MOF-808 functionalized with three 3,4-BHBA ligands in the equatorial plane. We show the IR spectra of the three configurations considered in this work.

References

(S1) Petříček, V.; Dušek, M.; Palatinus, L. Crystallographic computing system JANA2006: general features. *Z. Kristallogr. - Cryst. Mater.* **2014**, *229*, 345–352.

(S2) Furukawa, H.; Gandara, F.; Zhang, Y.-B.; Jiang, J.; Queen, W. L.; Hudson, M. R.; Yaghi, O. M. Water adsorption in porous metal–organic frameworks and related materials. *J. Am. Chem. Soc.* **2014**, *136*, 4369–4381.

(S3) Dippel, A.-C.; Liermann, H.-P.; Delitz, J. T.; Walter, P.; Schulte-Schrepping, H.; Seeck, O. H.; Franz, H. Beamline P02.1 at PETRA III for high-resolution and high-energy powder diffraction. *J. Synchrotron Radiat.* **2015**, *22*, 675–687.

(S4) Basham, M.; Filik, J.; Wharmby, M. T.; Chang, P. C. Y.; El Kassaby, B.; Gerring, M.; Aishima, J.; Levik, K.; Pulford, B. C. A.; Sikharulidze, I.; Sneddon, D.; Webber, M.; Dhesi, S. S.; Maccherozzi, F.; Svensson, O.; Brockhauser, S.; Náray, G.; Ashtona, A. W. Data analysis workbench (DAWN). *J. Synchrotron Radiat.* **2015**, *22*, 853–858.

(S5) Kresse, G.; Furthmüller, J. Efficient Iterative Schemes for Ab Initio Total-Energy Calculations Using a Plane-Wave Basis Set. *Phys. Rev. B* **1996**, *54*, 11169.

(S6) Blöchl, P. E. Projector Augmented-Wave Method. *Phys. Rev. B* **1994**, *50*, 17953.

(S7) Kresse, G.; Joubert, D. From Ultrasoft Pseudopotentials to the Projector Augmented-Wave Method. *Phys. Rev. B* **1999**, *59*, 1758.

(S8) Perdew, J. P.; Burke, K.; Ernzerhof, M. Generalized Gradient Approximation Made Simple. *Phys. Rev. Lett.* **1996**, *77*, 3865.

(S9) Grimme, S.; Antony, J.; Ehrlich, S.; Krieg, H. A Consistent and Accurate Ab Initio Parametrization of Density Functional Dispersion Correction (DFT-D) for the 94 Elements H-Pu. *J. Chem. Phys.* **2010**, *132*, 154104.

(S10) Furukawa, H.; Gándara, F.; Zhang, Y.-B.; Jiang, J.; Queen, W. L.; Hudson, M. R.; Yaghi, O. M. Water Adsorption in Porous Metal–Organic Frameworks and Related Materials. *J. Am. Chem. Soc.* **2014**, *136*, 4369–4381.

(S11) Wilson Jr., E. B.; Decius, J. D.; Cross, P. C. *Molecular Vibrations*; McGraw-Hill, 1955.

(S12) Woodward, L. A. *Introduction to the Theory of Molecular Vibrations and Vibrational Spectroscopy*; Oxford University Press, 1972.

(S13) Romero-Muñiz, C.; Paredes-Roibás, D.; Hernanz, A.; Gavira-Vallejo, J. M. A Comprehensive Study of the Molecular Vibrations in Solid-State Benzylic Amide [2]Catenane. *Phys. Chem. Chem. Phys.* **2019**, *21*, 19538–19547.

(S14) Giannozzi, P.; Baroni, S. Vibrational and Dielectric Properties of C_{60} from Density-Functional Perturbation Theory. *J. Chem. Phys.* **1994**, *100*, 8537–8539.

(S15) Baroni, S.; de Gironcoli, S.; Dal Corso, A.; Giannozzi, P. Phonons and Related Crystal Properties from Density-Functional Perturbation Theory. *Rev. Mod. Phys.* **2001**, *73*, 515–562.

(S16) Gajdoš, M.; Hummer, K.; Kresse, G.; Furthmüller, J.; Bechstedt, F. Linear Optical Properties in the Projector-Augmented Wave Methodology. *Phys. Rev. B* **2006**, *73*, 045112.
